# Beware the black box: investigating the sensitivity of FEA simulations to modelling factors in comparative biomechanics

**DOI:** 10.7717/peerj.204

**Published:** 2013-11-05

**Authors:** Christopher W. Walmsley, Matthew R. McCurry, Phillip D. Clausen, Colin R. McHenry

**Affiliations:** 1 Department of Anatomy and Developmental Biology, School of Biomedical Sciences, Monash University, Melbourne, Victoria, Australia; 2 School of Engineering, University of Newcastle, Newcastle, New South Wales, Australia; 3 Geoscience, Museum Victoria, Melbourne, Victoria, Australia

**Keywords:** Finite element analysis, Biomechanics, Sensitivity analysis, Crocodiles, Comparative biomechanics, FEA, Computational biomechanics, Comparative analysis, Scaling, Material properties

## Abstract

Finite element analysis (FEA) is a computational technique of growing popularity in the field of comparative biomechanics, and is an easily accessible platform for form-function analyses of biological structures. However, its rapid evolution in recent years from a novel approach to common practice demands some scrutiny in regards to the validity of results and the appropriateness of assumptions inherent in setting up simulations. Both validation and sensitivity analyses remain unexplored in many comparative analyses, and assumptions considered to be ‘reasonable’ are often assumed to have little influence on the results and their interpretation.

Here we report an extensive sensitivity analysis where high resolution finite element (FE) models of mandibles from seven species of crocodile were analysed under loads typical for comparative analysis: biting, shaking, and twisting. Simulations explored the effect on both the absolute response and the interspecies pattern of results to variations in commonly used input parameters. Our sensitivity analysis focuses on assumptions relating to the selection of material properties (heterogeneous or homogeneous), scaling (standardising volume, surface area, or length), tooth position (front, mid, or back tooth engagement), and linear load case (type of loading for each feeding type).

Our findings show that in a comparative context, FE models are far less sensitive to the selection of material property values and scaling to either volume or surface area than they are to those assumptions relating to the functional aspects of the simulation, such as tooth position and linear load case. Results show a complex interaction between simulation assumptions, depending on the combination of assumptions and the overall shape of each specimen. Keeping assumptions consistent between models in an analysis does not ensure that results can be generalised beyond the specific set of assumptions used. Logically, different comparative datasets would also be sensitive to identical simulation assumptions; hence, modelling assumptions should undergo rigorous selection. The accuracy of input data is paramount, and simulations should focus on taking biological context into account. Ideally, validation of simulations should be addressed; however, where validation is impossible or unfeasible, sensitivity analyses should be performed to identify which assumptions have the greatest influence upon the results.

## Introduction

### Aims

Here we investigate the sensitivity of models in a broad scale comparative Finite Element (FE) dataset to different values of several modelling factors, to determine the extent by which the pattern of results is changed by the choice of different input values. The specific focus is on factors associated with material properties, scaling, linear load cases, and bite position.

Our approach is to make use of a previously compiled comparative dataset, which drew conclusions relating to form and function in extant crocodilians (Walmsley et al., 2013). As in the previous study, we simulate biting, shaking, and twisting feeding behaviours, which are typically used by crocodilians to process prey items. We explore many of the modelling factors inherent in the growing body of comparative biomechanical studies, and explicitly test the extent to which these factors influence, or change the pattern of results.

### Factors affecting FE analysis

Finite Element Analysis (FEA) is a computational technique commonly used in engineering disciplines, whereby complex structures are discretised in order to approximate their mechanical response (behaviour) to applied loads. In recent years FEA has become increasingly prevalent in the fields of comparative biomechanics (McHenry et al., 2006; Oldfield et al., 2012; Walmsley et al., 2013), paleontology (Degrange et al., 2010; McHenry, 2009; McHenry et al., 2007; Tseng & Wang, 2010; Wroe, 2008; Wroe et al., 2013), biology (Dumont, Piccirillo & Grosse, 2005; Wroe et al., 2008), medicine (Chen et al., 2012; Omasta et al., 2012), and anthropology (Wroe et al., 2010), as improvements in computational capabilities mean lower entry level costs for researchers. In the context of comparative biomechanics FEA offers a number of advantages:

1. Biological structures included in comparative analyses often differ in size and shape, whilst structure-function questions typically focus on the role of shape. In Finite Element (FE) models, differences between specimens can be easily standardized through scaling (Dumont, Grosse & Slater, 2009; McHenry, 2009; Snively, Anderson & Ryan, 2010; Tseng, 2008; Walmsley et al., 2013).
2. Experiments can be quickly changed to test new hypotheses, simply by changing boundary conditions and loading.
3. Experimental time is greatly reduced, and since simulations are digital many more tests can be performed on a single specimen than would be feasible working with live animals or *ex vivo* specimens.
4. Hypotheses on form and function implications for extinct taxa can be tested (McHenry et al., 2007; Oldfield et al., 2012; Plotnick & Baumiller, 2000; Snively & Theodor, 2011; Tseng, 2008; Wroe et al., 2007a).
5. When combined with mesh deformation/warping (O’Higgins et al., 2011; Parr et al., 2012), purely theoretical morphotypes can be generated to help tease out the important features of shape that effect the structural response.

Despite the many advantages of FEA, there are limitations to the conclusions that can be drawn from the results (Rayfield, 2007). Finite element models are complex and informative simulations require deliberate choices for multiple factors (listed below, and here termed **modelling factors**). In many instances, biologically relevant empirical data for each factor are lacking and researchers necessarily make assumptions about realistic/plausible values to use as input variables for these modelling factors (McHenry et al., 2006).

The goal of many comparative analyses is to discover the pattern of differences in biomechanical performance between different models; that is, the relative order of the models’ performance under specific loads (e.g., strongest to weakest) and the degree by which they vary. While sufficient accuracy is critical in mechanical and biomedical engineering, for many comparative biomechanical studies the accuracy of absolute results is not required as long as the interspecific pattern of results resembles the actual biological pattern (McHenry et al., 2007; Oldfield et al., 2012; Parr et al., 2012; Rayfield, 2005; Tseng, 2008; Walmsley et al., 2013; Wroe et al., 2007a; Wroe et al., 2010). Whether the FEA results do reflect reality can only be examined if the results of the analysis can be compared with empirical data, a process termed validation. Although validation data has been used in a number of biological FE analyses (Bright, 2012; Bright & Rayfield, 2011a; Bright & Rayfield, 2011b; Gröning et al., 2009; Kupczik et al., 2007; Liu et al., 2012; Metzger, Daniel & Ross, 2005; Panagiotopoulou et al., 2010; Panagiotopoulou et al., 2012; Rayfield, 2011; Ross et al., 2005; Strait et al., 2005; Tsafnat & Wroe, 2011), the data required to validate models are difficult to obtain. Many comparative biomechanical analyses are thus run without validation (McHenry, 2009; McHenry et al., 2006; McHenry et al., 2007; Oldfield et al., 2012; Walmsley et al., 2013; Wroe et al., 2007a; Wroe et al., 2008). Combined with the lack of data on realistic values for modelling factors, this creates a degree of uncertainty about the validity of the results. In many cases, researchers assume (either explicitly or implicitly) that the precise value of input variables for modelling factors will not alter the pattern of results, as these values are kept constant across the models in the analysis, and the results obtained will be a valid reflection of the pattern. Whilst this is a logically plausible approach, it is seldom tested.

In the absence of the required empirical data to validate FE models, the sensitivity of results to the choice of input values for modelling factors can be explored through sensitivity analysis. In such an analysis, the input value for one factor is varied across models, while all other values are held constant; thus the effect upon the pattern of results can be quantified. If the pattern of results does not change markedly for different values, then the analysis is deemed relatively insensitive to the precise values chosen for that modelling factor. Where this is the case, the assumption – that the results of a comparative analysis can be informative, even in the absence of empirical data on modelling factors and absence of model validation – remains logically plausible (although still untested). If, however, the pattern of results is strongly affected by the precise values used for modelling factors, then the analysis is sensitive to input parameters and its results are only informative if input parameters are founded upon empirical data.

Investigations into the sensitivity of FEA simulations have been performed in relation to a number of different modelling factors. These investigations have targeted input values associated with scaling (Dumont, Grosse & Slater, 2009), material properties (Bright & Rayfield, 2011b; Cox et al., 2011; Gröning, Fagan & O’Higgins, 2012; Kupczik et al., 2007; Panagiotopoulou et al., 2010; Porro et al., 2011; Reed et al., 2011; Strait et al., 2005; Tseng et al., 2011; Wroe et al., 2008), muscle activation (Fitton et al., 2012; Ross et al., 2005; Tseng et al., 2011), sutures (Bright, 2012; Kupczik et al., 2007; Porro et al., 2011; Reed et al., 2011; Wang et al., 2010), bite position (Cox et al., 2011; Fitton et al., 2012; Porro et al., 2011; Wang et al., 2010), ligaments (Gröning, Fagan & O’Higgins, 2012; Gröning, Fagan & O’Higgins, 2011; Wood et al., 2011), mesh density (Bright & Rayfield, 2011a; Tseng et al., 2011), mesh warping (O’Higgins et al., 2011), jaw joint constraint (Gröning, Fagan & O’Higgins, 2012; Tseng et al., 2011), orientation of muscle force (Bright & Rayfield, 2011b; Cox et al., 2011; Gröning, Fagan & O’Higgins, 2012; Grosse et al., 2007), muscle loading application (Grosse et al., 2007; Kupczik et al., 2007; Wroe et al., 2008), FEM element type (Bright & Rayfield, 2011a; Dumont, Piccirillo & Grosse, 2005), and subcortical geometries (Panagiotopoulou et al., 2010). This growing body of literature has helped to identify those modelling factors which most affect simulation results (information which is invaluable to comparative studies where validation is unfeasible or impossible). However, the majority of sensitivity analyses to date involve a single specimen; there are limited instances of sensitivity analyses involving either multiple specimens of one species (Kupczik et al., 2007), or multiple species (Cox et al., 2011). As an important goal of many comparative analyses is to ascertain the pattern of relative biomechanical performance between taxa, multi-factorial, multi-species sensitivity analyses allow us to assess how suitable FEA is for comparative studies in the absence of validation.

### Modelling factors

Modelling factors in comparative FEA are specific aspects of model set-up that can influence results in comparative simulations. Common modelling factors include, but are not limited to: scaling, material properties, simulated feeding behaviour, linear versus non-linear load cases, sutures, bite position, muscle activation schemes, muscle proportions, number of muscles, constraints, and how muscles are modelled in the FE simulation. Each of these factors can often be implemented in a number of different ways; for example, muscles are typically modelled either as beams or as point loads, and both of these implementations contain subsets of options, such as beam geometry and directionality of the point load. In addition to the numerous combinations in which they can be sensibly assembled, the cascade of assumptions within each modelling factor leads to a very large parameter space, which can potentially produce appreciably different results if sensitivity to these is high.

Exploring this parameter space is logistically complex. In the absence of empirical data that can be used to select realistic input values for the various factors, exploration of parameter space provides a sensitivity analysis but does not necessarily improve model accuracy. In this paper we present a comprehensive sensitivity analysis of the following five modelling factors, which are each specifically relevant to questions about skull optimisation (minimized stress/strain) for given feeding scenarios in crocodilians.

#### Scaling

Biological structures typically vary in shape and size, and for questions relating to the function of shape, size becomes a confounding factor that needs to be removed from the results. Most commonly this is achieved by scaling each specimen to some common measure of overall size, usually volume (McHenry, 2009; Oldfield et al., 2012; Tseng, 2008; Walmsley et al., 2013) or surface area (Dumont, Grosse & Slater, 2009), and far less frequently to a linear measure such as length (chosen for ecological comparability) (Close & Rayfield, 2012; Snively, Anderson & Ryan, 2010). The selection of appropriate scaling parameters for a comparative study is important and often dependent on the scope and design of the specific question being addressed (Dumont, Grosse & Slater, 2009).

#### Material properties

In comparative biomechanics studies, material properties can be simulated as heterogeneous (McHenry et al., 2007; Snively & Theodor, 2011; Tseng et al., 2011; Tseng & Wang, 2010) or homogeneous (Oldfield et al., 2012; Tseng & Wang, 2010; Walmsley et al., 2013), and since accurate information is often unavailable, or largely unknown, specific data is often appropriated from other better known taxa. Studies including extinct taxa often use homogeneous material properties (Oldfield et al., 2012; Snively & Theodor, 2011; Tseng & Wang, 2010; Wroe et al., 2010), as taphonomy often alters the structure and density of the preserved bone, although heterogeneous properties have been applied to fossils with exceptional preservation (McHenry et al., 2007; Wroe, 2008). In rare cases where geometric locations of cortical and spongy bone can be approximated, quasi-heterogeneous properties, consisting of bulk properties of spongy and cortical bone have been applied in fossil taxa (Strait et al., 2009); this practice is far more common in extant taxa (Bright & Rayfield, 2011b; Panagiotopoulou et al., 2010) since these regions can be more readily identified.

#### Feeding behaviour

The feeding behaviour selected in comparative simulations is typically chosen based on the specific questions and hypotheses that the study aims to address. While feeding behaviour is not strictly a ‘modelling factor’ on its own, it defines the context (the problem definition) used to determine appropriate boundary and loading conditions, and represents the combination of the assumptions used in the simulations. Examples of different feeding behaviours commonly simulated include, but are not limited to, both bilateral (Tseng & Wang, 2010; Walmsley et al., 2013) and unilateral (Tseng & Wang, 2010) biting, shaking (McHenry, 2009; Walmsley et al., 2013), twisting (McHenry, 2009; Walmsley et al., 2013), and pull back (Moreno et al., 2008; Wroe, 2008).

#### Linear load case combinations

Linear Load Case combinations (LLCs) are often used to adjust the relative loading of simulations to comparable measures. For example, in simulations involving biting there are often two logically plausible options for simulations: (1) Simulate all specimens biting at their maximal muscle force (McHenry et al., 2007), and (2) simulate all specimens biting with the same ‘resultant’ bite force (Walmsley et al., 2013). Generally the selection of either (1) or (2) is dependent on the question being addressed: (1) addresses which specimen is capable of generating the largest forces, and (2) addresses which specimen performs better (or are better optimised) for a particular load. Similarly different LLCs have been used to analyse other behaviours such as shake and twist feeding (Walmsley et al., 2013), pull back (Moreno et al., 2008; Wroe, 2008), and unilateral biting (Clausen et al., 2008; Ross et al., 2005).

#### Bite position

Selection of bite position is often a key assumption used in simulations, and is typically chosen such that it represents a functionally similar location for all species in the dataset (i.e., towards the front, mid, or back of the tooth row). Determining which bite position is the most appropriate for a particular simulation typically depends on the specific feeding behaviour being simulated, and should be based upon observational data. For example, in crocodilian taxa, large prey is often reduced for consumption by either shaking or twisting (Taylor, 1987); for each of these, anecdotal information suggest that prey are held in the front part of the jaws, although quantitative data are once again lacking. Therefore, in this context it may be more appropriate to compare these feeding types at either front or mid positions. Conversely, animals that tend to feed on hard prey items may be more likely to bite using their rear teeth than those at the front, and thus should be compared at their rear positions (Tseng & Binder, 2010).

## Methods

To compare how multi-dimensional variation of input parameters affects the pattern of results in an interspecific comparative biomechanics analysis, we used seven high resolution (>1 million elements) FE models of crocodilian skulls. These models formed the basis for a previous study that investigated the relationship between mandible shape and biomechanical performance in crocodilians (Walmsley et al., 2013). The seven species modelled were *Crocodylus intermedius* (Ci), *Crocodylus johnstoni* (Cj), *Crocodylus moreletii* (Cm), *Crocodylus novaeguineae* (Cng), *Mecistops cataphractus* (Mc), *Osteolaemus tetraspis* (Ot), and *Tomistoma schlegelii* (Ts).

### FEA models

For this analysis many methodological aspects (particularly those relating to data acquisition, CT processing, and surface/solid mesh generation) are identical to the previous study (Walmsley et al., 2013). In summary: CT data was processed in MIMICS v11 (MATERIALISE, Belgium), surface meshes of the cranium and mandible were optimised before forming the foundation to generate suitable solid meshes using Harpoon (SHARC), and FE simulations were performed using Strand7 (www.strand7.com).

High-resolution finite element model construction was based on previously published protocols (Bourke et al., 2008; Clausen et al., 2008; McHenry et al., 2007; Moreno et al., 2008; Wroe et al., 2007a); see Walmsley et al. (2013) for specific details. In the present study, we varied the parameters (described below in detail) of several modelling factors: **Material properties**: models are simulated with either isotropic-heterogeneous or -homogeneous material properties; **Scaling**: models are scaled to a consistent volume, surface area, or length; **Feeding behaviour**: models are loaded to simulate biting, shaking, and twisting feeding behaviours; **Linear load case combinations**: loads are scaled to 2 metrics per feeding behaviour (see below for details); **Bite position**: simulations are performed at front, mid, and back bite positions. Each of these variables is altered whilst holding all others constant, allowing all possible combinations of variables to be investigated across the seven species simulated. Specific feeding behaviours are strictly functional variables and are not considered to be a target of this sensitivity analysis – it is nonsensical to investigate strength under twisting as an indicator of strength under biting – but these load cases do increase the parameter space investigated in this study by a factor of three, and including them gives some insight into how sensitivity to a modelling factor is affected by functionally different loading conditions. Whilst the breadth of modelling factors explored here is extensive, it is by no means complete; for example, our simulations do not account for the influence of structures such as sutures, which have an important effect on biomechanics (Bright, 2012; Kupczik et al., 2007; Porro et al., 2011; Reed et al., 2011; Wang et al., 2010).

#### Material properties

Isotropic heterogeneous material properties were calculated for each tetrahedral element based on the corresponding Hounsfield Unit (HU) attenuation of the voxels in 3D space. Material properties were applied to each model using MIMICS v11, and values are defined according to a combination of empirically derived values of bovine femur (McHenry et al., 2007) and a slightly modified linear relationship derived from Hounsfield values for water (0 HU) and air (−1000 HU). Since the range of HU varies between the mandible and cranium (Table 1), each specimen had 50 different isotropic material properties applied for the cranium and the mandible respectively; 100 in total for each model. Bone is often found to be anisotropic (Currey, 2002; Zapata et al., 2010), and anisotropic material properties have been described in the mandible of the extant crocodilian *Alligator mississippiensis*, in which the mandible is stiffest about its long axis (Zapata et al., 2010). Although the effect that anisotropy has on simulations of the alligator mandible has been investigated (Porro et al., 2011; Reed et al., 2011), in the present sensitivity study only isotropic materials are addressed.

**Table 1:** HU range for each specimen. Note the differences in the range between the mandible and cranium occurs for all species with the exception of *C. novaeguineae*, due to separate scans of the cranium and mandible.

	Hounsfield Unit (HU) Range	
Taxon	Cranium	Mandible
*Osteolaemus tetraspis*	−724 to 2339	−719 to 2248
*Crocodylus moreletii*	−1018 to 2848	−975 to 2724
*Crocodylus novaeguineae*	−1024 to 3071	−1024 to 3071
*Crocodylus intermedius*	−1024 to 1829	−1024 to 2097
*Crocodylus johnstoni*	−1024 to 2260	−1024 to 2264
*Mecistops cataphractus*	−665 to 2022	−596 to 2023
*Tomistoma schlegelii*	−742 to 2327	−704 to 2109

Isotropic homogeneous material properties are calculated such that mass is conserved between heterogeneous and homogeneous models of *M. cataphractus* (see Walmsley et al., 2013); this average value of bone density and elastic (Young’s) modulus was applied to all other models in this study.

Models with an isotropic heterogeneous property set are hereafter dubbed ‘HET’, whilst ‘HOM’ denotes models with isotropic homogeneous property sets.

#### Scaling

In our previous analysis (Walmsley et al., 2013), all models were rescaled to volume only. Here models were rescaled according to three criteria: (a) all models had the same cranial + mandible volume (for the tetrahedral ‘brick’ elements) as the *M. cataphractus* model, (b) all models had the same cranial + mandible surface area (dubbed ‘surface’ from here on and calculated from brick elements) as the *M. cataphractus* model, and (c) all models had the same length (measured from the jaw hinge axis to the most rostral midline point of the premaxillae). Muscle beam pre-tensions for each model are scaled according to the re-scaled (volume, surface, and length) size (Walmsley et al., 2013). For each criterion, the parameter value (volume, surface, length) for each unscaled model was measured in Strand7, and models were then rescaled accordingly using Strand7’s ‘rescale’ command.

#### Feeding behaviour

Load cases are defined as described in Walmsley et al. (2013), and reflect the three broad categories of behaviours used by crocodilians to kill and process large prey: biting (jaw adduction), shaking (where prey is held in the jaws and rapidly accelerated from side to side), and twisting (where prey is held in the jaws while the crocodile spins rapidly around its own long axis (Taylor, 1987)). These are functionally different behaviours and, as explained above, do not constitute parameters targeted for the sensitivity analysis, although they do increase parameter space. As in Walmsley et al. (2013), biting load cases are simulated by restraining the teeth at relevant bite points (see below) and applying pre-tension loads to the adductor muscle beams. Shaking is simulated by applying a direct force to each of the teeth involved with a given bite position, whilst twisting is simulated by restraining the teeth relevant to a specific bite position in space and applying a torque about the long axis of the skull to the caudal-most node on the occipital condyle. For a detailed description of how beam pre-tension, shake force, and torque was calculated see McHenry (2009), and Figures 12, 14 and 15 from Walmsley et al. (2013).

#### Bite positions

For each combination of scaling, material properties, and load case, three bite positions where assessed. Each bite position involved four teeth. Front bites involved the largest teeth in the premaxillary tooth row (the 4th premaxillary teeth on the left and right sides) and their closest apposing teeth in the lower jaw. Mid and rear bites involved the 5th and 10th maxillary teeth and their closest apposing teeth in the lower jaw respectively.

#### Linear load case combinations (LLCs)

These are simulated at front, mid, and back bite positions for each re-scaled (volume, surface, and length) size, for both HET and HOM material properties.

Biting is simulated at each rescaled size by adjusting muscle forces to (1) the $\frac{2}{3}$ power of the ratio of ‘scaled volume’ to the ‘original volume’ (‘no linear load case’, or ‘NoLLC’; see Walmsley et al. (2013), and (2) so that the resultant bite force was equivalent to the bite force from the *M. cataphractus* model (‘tooth equals tooth’, or ‘TeT’). Note that adjusting the muscle forces to the $\frac{2}{3}$ power of the ratio of ‘scaled volume’ to ‘original volume’ results in all species models biting at their maximal calculated muscle force at that rescaled size (McHenry, 2009; McHenry et al., 2007).

Shaking is simulated so that (1) the magnitude of the shake force was equivalent to that calculated for *M. cataphractus* (‘tooth equals tooth’, or ‘TeT’), and (2) the ratio of outlever-length (perpendicular distance from the jaw hinge axis to the centre of mass of the prey item) to shake force remained constant between models (‘equal lever arm’, or ‘ELA’). Keeping this ratio constant between models has the effect of simulating each model shaking a prey item of equal mass at the same frequency. If each model shakes with the same force (e.g., when scaled to volume), the small differences between outlever-length would mean that either some models are shaking prey of the same mass at a higher frequency, or that they are shaking prey of larger mass at the same frequency.

Twisting is simulated so that (1) the magnitude of the twisting force was equivalent to that calculated for *M. cataphractus* (‘moment equals moment’, or ‘MeM’), and (2) so that the ratio of skull width to twisting force remains constant between models (‘ELA’).

### Data collection and presentation

We collected, assessed and here present data in multiple formats to ascertain the degree that multi-dimensional variation of input parameters (for common modelling factors) has on the results and their interpretation. In brief (outlined in detail below), the presented formats are:

• *Signal:***qualitative** visual comparison between **pairs** or **triplets** of *sets* within **one** species.
• *Rank:***qualitative** comparison between **pairs** of *conditions* between **multiple** species.
• *Percentage Difference and Mean Percentage Difference:***quantitative** comparison between **pairs** of *conditions* within **one** species.
• *Pattern and Standardised Pattern:***quantitative** comparison between **all** *conditions*, for **multiple** species.
• *Standardised Pattern Difference (SPD):***quantitative** comparison between *sets*, for **multiple** species, and allowing comparison of **qualitatively- vs quantitatively-ordered** data.
• *Shape correlations:***quantitative** comparison between shape differences and *set* differences, for **multiple** species.

As in Walmsley et al. (2013) the results assessed here are the 95% von Mises strain values; this 95% von Mises strain constitutes the largest elemental (individual brick) value of strain in the model if the highest 5% of all elemental values are ignored. It is important to note that measuring strain in this way only accounts for the magnitude of strain; it compares the magnitude of upper strain values but does not include any information on the type of strain (i.e., compressive or tensile), or upon the location of that strain within the anatomical structure. Each individual result is a combination of specific values for each parameter; we use *condition* to describe that combination of parameters. In total 108 unique *conditions* exist for each species model, each describing the type of scaling (volume, surface, or length), bite position (front, mid, or back), feeding type (bite, shake, or twist), material properties (HET or HOM), and specific LLCs (one of a possible two per feeding type) used in an individual simulation. A *set* is used to describe an arbitrarily ordered group of *conditions* ($X=\left\{{x}_{1},{x}_{2},\ldots ,{x}_{n}\right\}$) with a common parameter. When comparing between two *sets*, the number of *conditions* in each *set* depends upon the number of values (parameter options) for the modelling factor; where there are two parameter options (i.e., for material properties and LLC) the number of *conditions* in a *set* is 54, and where there are three parameter options (i.e., for scaling, feeding type, and bite position) there are 36 *conditions* per *set*. Thus, a ‘volume’ *set* would include all 36 *conditions* where the model is scaled to volume, and a ‘HOM’ *set* includes all 54 *conditions* where the model has isotropic homogeneous material properties.

#### Signal

**Figure 1:**
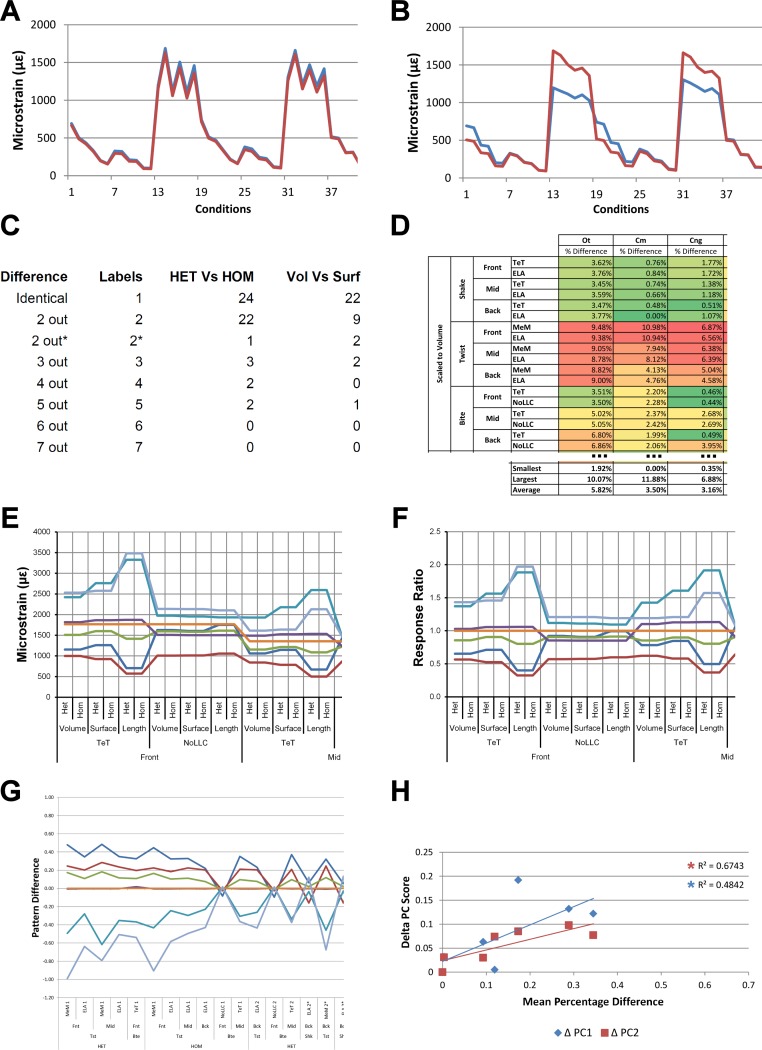
Data collection and visualisation. Data presented here is used only to illustrate and summarise how results are presented, interpreted, and analysed. Response is plotted as a *signal* for different *sets* (a ‘*set’* is an arbitrarily ordered group of *conditions* with a common parameter) showing good (A) and poor (B) correlation between input *conditions* for an individual species model. (A) corresponds to a HET vs HOM comparison for *O. tetraspis* (see Fig. 2A), while (B) corresponds to a Linear Load Case comparison for *O. tetraspis* (see Fig. 8A). (C) Predictive rank of species models between comparison *sets*. Labels are used as shorthand to indicate how well rank predictions correlate between input *conditions*, low numbers indicate good correlation, while high indicates poor correlations. See Table 3 for specific details, and Figs. 5, Figs. S2, S6–S8, S10, S14–S16 for label implementation. (D) Absolute percentage difference between the response of each species model (indicated above columns) for comparison *sets*; green to red shading indicates low to high values. Here (D) corresponds to a HET vs HOM comparison snipped from Fig. S1; note that the *conditions* from top to bottom in (D) also correspond to those ordered left to right in *signal* (A). This is consistent for all compared modelling factors; e.g. for scaling comparisons the order is consistent between *signal* (Fig. 7) and percentage differences (Figs. S3–S5). (E) Charts of pattern, plotting strain response of individual species models (coded by colour) to individual *conditions*. (F) Charts of standard pattern plot the ratio (‘Response ratio’) of strain response in each species (ε_*sp*_) model (coded by colour) to strain in *M. cataphractus* (ε_*Mc*_) for individual *conditions*, i.e., ε_*sp*_/ε_*Mc*_. (G) Charts of standard pattern difference, where the difference in standard pattern is taken between *pairs* of *sets* under comparison. (H) Interspecies shape difference (ΔPC1 and ΔPC2) plotted against mean percentage difference to determine if differences between comparison *sets* correlate with shape differences.

*Signal* is plotted as the microstrain value for each *condition*, with the *set* of *conditions* for each value plotted along the *x* axis. For *signal*, all *sets* for a modelling factor can be plotted simultaneously (e.g., Figs. 1A and 1B). This gives a chart that superficially resembles a signal trace. By treating the strain response of each specimen as a *signal* to a *set* of unique *conditions*, differences between the variables for each modelling factor can be compared at a holistic level, and the closer one *signal* follows (or tracks) to another, the less influence that modelling factor has upon the results (Figs. 1A and 1B).

#### Rank

Ranking specimens based on strain response is often used to draw conclusions about their relative performance within a study group (McHenry et al., 2006; Oldfield et al., 2012). For each *condition*, the ranked order of the models (from lowest strain to highest strain) was compared between pairs of *sets*, and scored accordingly to whether 0, 2, 3, 4, 5, 6 or 7 species models had different ranks between those *sets* (note that it is impossible for there to be only 1 difference in ranking – for convenience, ‘0’ difference rankings are scored as 1 in figures – so here a score of 1 indicates identical predictions of rankings). For the material properties and LLC modelling factors, this gave 54 pairwise comparisons of ranked order, and 36 for the remaining modelling factors. For a pair of *sets*, if scores were predominantly 1 or 2, then ranked orders were very similar and those values for that modelling factor were deemed to have only a small effect upon the qualitative pattern of results. Scores that were predominantly 4, 5, 6, or 7 had quite different rankings, and those values were deemed to have significant qualitative effects (Fig. 1C).

#### Percentage difference and mean percentage difference

For each pair of *conditions* within a comparison of *sets*, percentage differences are calculated as the absolute difference in strain response for each model as a percentage of the larger value in that pair. The mean value of this figure for all of the *conditions* in a *set* is then calculated for each species model (Fig. 1D).

#### Pattern and standardised pattern

A plot of strain values for all of the species models across all *conditions* provides a graphical representation of quantitative *pattern*, in addition to providing a visual representation (Fig. 1E). If strain values are standardised to a benchmark species, the shape of the qualitative (and quantitative) pattern is maintained and is clearer in the chart. We use the *Mecistops cataphractus* model as the benchmark species model, so that the strain response of each species to load is plotted as ε_*species*_/ε_*Mc*_ (where ε is strain, and ε_*Mc*_ denotes values for *M. cataphractus*) for each *condition*, providing a chart of *standardised pattern* of results (Fig. 1F).

#### Standardised pattern difference (SPD)

In a qualitative comparison of pattern, pairs of *conditions* are judged to be similar if the rank of a species model is similar across that pair, but rankings also provide an index of pattern similarity across the seven difference species models. Values of percentage difference provide a quantitative version of this test, but are limited to within-species model pairwise comparisons. To provide an index of the degree by which pattern across species varies quantitatively for each pair of *conditions*, we take the difference between standardised pattern values for each species across the *conditions* in a *set*. This number – the *standardised pattern difference* (SPD) – provides a quantitative index of the degree to which the pattern of results is similar across *condition* pairs. An advantage of this index is that those differences can be summed across species models for each *condition*, giving a total standardised pattern difference for each pair of *conditions* in a *set* (Fig. 1G).

Within each *set*, it is possible to order the conditions according to the degree of qualitative or quantitative variation in the pattern; for example, conditions that have a low score of difference in rankings can be shown to the left of the *x* axis, with conditions plotted towards the right representing sequentially higher degrees of differences in ranked order. Likewise, conditions can be ordered along the *x* axis according to a quantitative measure, such as total standardised pattern difference. The similarity in the order of conditions within a *set* when ordered by qualitative vs quantitative scores provides an additional opportunity to evaluate the sensitivity of the analysis to modelling factors. If these are similar for a modelling factor, then the degree of sensitivity is qualitatively and quantitatively similar. We evaluate by comparing visual plots of standardised pattern difference data, ordered by (1) rank, and (2) total standardised pattern difference.

#### Shape correlations

Here we assess whether the difference between comparison *sets* is a function of interspecific differences in shape. Using principal component values (PC1 and PC2) from Walmsley et al. (2013) we calculate the difference in shape between all species models to that of *M. cataphractus* for PC1 and PC2, yielding a ΔPC1 and ΔPC2 value for each species model (Table S1); these are essentially a measure of relative difference in the shape of each species to that of *M. cataphractus*. As in Walmsley et al. (2013) only the first two principal components are used, since between them they account for 92% of shape variation (66% PC1, 26% PC2). As summarised in Walmsley et al. (2013), variation in shape along each PC axis was quantified against the following 4 morphological measures: mandibular length (L), symphyseal length (SL), inter-rami angle (A), and mandibular width (W). PC1 correlated best with SL followed by W, while PC2 correlated best with A followed closely by both SL and W (for a comprehensive description of shape variation along each PC axes see Figures 18 and 19 in Walmsley et al. (2013)). These are then plotted against the mean percentage difference values of each species for each comparison *set* to determine if those differences are a function of shape (Fig. 1H).

## Results

### Material properties (isotropic HET vs isotropic HOM)

**Figure 2:**
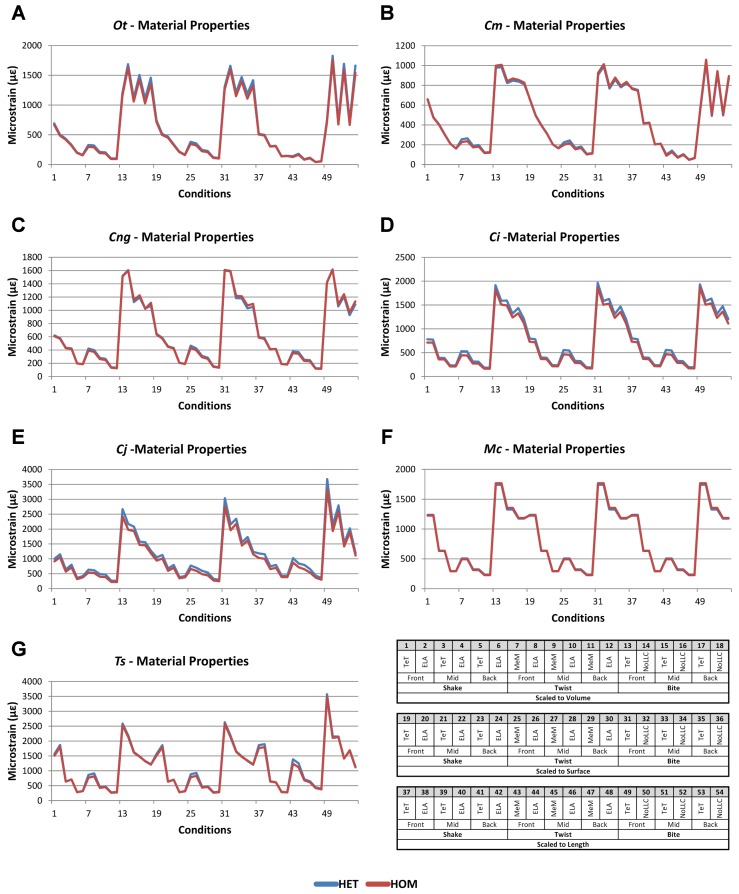
HET and HOM *signal* for simulation *conditions*. Simulation *conditions* are arbitrarily numbered from 1 to 54 (labelled bottom right); for each *condition* the response to HET material properties is graphed alongside the response to HOM material properties. TeT (‘tooth equals tooth’), NoLLC (‘no linear load case’), ELA (‘equal lever arm’), and MeM (‘moment equals moment’) each indicate the type of linear load case used in the simulation. Under biting, TeT simulates all species biting with identical ‘resultant’ bite force to *M. cataphractus*, while NoLLC simulates all species biting at their maximal muscle force. Under shaking, TeT simulates an identical magnitude of shake force to *M. cataphractus,* while ELA simulates shaking prey of identical mass at the same frequency. Under twisting, MeM simulates an identical magnitude of twisting force, while ELA simulates a constant ratio of skull width to twisting force between each species. Note that for all species the response to HET tracks very closely to HOM, and differences for *M. cataphractus* are almost indistinguishable. (A) Ot, *Osteolaemus tetraspis*, (B) Cm, *Crocodylus moreletii*, (C) Cng, *Crocodylus novaeguineae*, (D) Ci, *Crocodylus intermedius*, (E) Cj, *Crocodylus johnstoni*, (F) Mc, *Mecistops cataphractus*, (G) Ts, *Tomistoma schlegelii.*

Results for HET and HOM models closely match each other across all *conditions*, both qualitatively and quantitatively. The *signal* of HOM models tracks closely to HET (Fig. 2) and percentage differences between each pair of *conditions* were consistently low, averaging <6% for all species excluding *C. intermedius* and *C. johnstoni*, which each averaged ≈10% (Table 2 and Fig. S1). Between *conditions*, the greatest differences were for those that involved twisting, but mean percentage difference values remained below 10% (Table 2). Between species models the largest differences were for *C. johnstoni* and *C. intermedius*, while the smallest were for *M. cataphractus* and *C. novaeguineae* (Fig. S1).

**Table 2:** Percentage differences summary.

			Ot	Cm	Cng	Ci	Cj	Mc	Ts	Average
**Het vs Hom**	Volume	Shake	3.61%	0.58%	1.27%	8.56%	10.31%	0.57%	1.70%	**3.80%**
		Twist	9.08%	7.81%	5.97%	13.47%	16.67%	3.15%	7.02%	**9.03%**
		Bite	5.12%	2.22%	1.79%	6.32%	7.67%	1.40%	1.29%	**3.69%**
	Surface	Shake	3.70%	0.64%	1.39%	8.56%	11.12%	0.57%	1.80%	**3.97%**
		Twist	8.93%	7.72%	6.01%	13.54%	16.70%	3.15%	7.03%	**9.01%**
		Bite	4.94%	2.14%	2.43%	6.28%	7.63%	1.40%	1.27%	**3.73%**
	Length	Shake	2.29%	0.59%	1.24%	8.69%	12.27%	0.57%	2.97%	**4.09%**
		Twist	8.89%	7.77%	5.99%	13.59%	16.63%	3.15%	7.02%	**9.01%**
		Bite	5.77%	1.99%	2.34%	5.98%	7.75%	1.40%	1.44%	**3.81%**
	**Average**		**5.82%**	**3.50%**	**3.16%**	**9.44%**	**11.86%**	**1.71%**	**3.50%**	
**LLC**	Volume	Shake	23.89%	25.20%	4.90%	0.40%	15.94%	0.00%	12.38%	**11.81%**
		Twist	2.13%	3.80%	5.65%	0.76%	3.44%	0.00%	5.38%	**3.02%**
		Bite	26.45%	2.31%	5.74%	16.57%	20.47%	0.00%	10.84%	**11.77%**
	Surface	Shake	27.71%	22.15%	7.81%	1.69%	10.20%	0.00%	11.70%	**11.61%**
		Twist	7.04%	7.77%	8.71%	2.17%	9.53%	0.00%	4.62%	**5.69%**
		Bite	18.60%	8.00%	1.14%	19.00%	30.56%	0.00%	12.38%	**12.81%**
	Length	Shake	2.90%	2.40%	2.08%	1.90%	2.85%	0.00%	3.54%	**2.24%**
		Twist	22.64%	28.47%	2.24%	2.27%	17.87%	0.00%	9.81%	**11.90%**
		Bite	58.12%	45.34%	13.09%	19.01%	42.89%	0.00%	35.56%	**30.57%**
	**Average**		**21.05%**	**16.16%**	**5.71%**	**7.08%**	**17.08%**	**0.00%**	**11.80%**	
**Volume vs Surface**	HET	Shake	4.83%	2.00%	2.84%	1.53%	3.53%	0.00%	0.57%	**2.18%**
		Twist	11.93%	9.89%	7.81%	3.64%	15.12%	0.00%	2.06%	**7.21%**
		Bite	4.93%	3.99%	2.36%	1.45%	6.40%	0.00%	0.84%	**2.85%**
	HOM	Shake	4.74%	1.94%	2.72%	1.52%	3.48%	0.00%	0.64%	**2.15%**
		Twist	12.08%	9.79%	7.76%	3.56%	15.08%	0.00%	2.06%	**7.19%**
		Bite	4.87%	3.95%	3.11%	1.48%	6.46%	0.00%	0.89%	**2.97%**
	**Average**		**7.23%**	**5.26%**	**4.43%**	**2.20%**	**8.34%**	**0.00%**	**1.18%**	
**Volume vs Length**	HET	Shake	16.92%	18.06%	2.83%	1.83%	8.63%	0.00%	7.83%	**8.01%**
		Twist	50.98%	53.29%	8.74%	4.13%	32.88%	0.00%	32.32%	**26.05%**
		Bite	23.40%	24.07%	4.30%	1.30%	14.58%	0.00%	14.37%	**11.72%**
	HOM	Shake	15.81%	17.92%	2.79%	1.69%	8.49%	0.00%	7.32%	**7.72%**
		Twist	50.89%	53.25%	8.77%	4.01%	32.90%	0.00%	32.33%	**26.02%**
		Bite	23.32%	23.99%	3.58%	1.65%	14.55%	0.00%	14.37%	**11.64%**
	**Average**		**30.22%**	**31.76%**	**5.17%**	**2.43%**	**18.67%**	**0.00%**	**18.09%**	
**Surface vs Length**	HET	Shake	20.63%	17.02%	5.56%	0.31%	6.08%	0.00%	7.62%	**8.17%**
		Twist	56.68%	48.29%	15.84%	0.51%	21.05%	0.00%	30.92%	**24.76%**
		Bite	26.81%	21.40%	6.53%	0.43%	9.10%	0.00%	13.71%	**11.14%**
	HOM	Shake	19.44%	16.92%	5.41%	0.17%	5.26%	0.00%	7.04%	**7.75%**
		Twist	56.67%	48.32%	15.83%	0.46%	21.11%	0.00%	30.93%	**24.76%**
		Bite	26.66%	21.36%	6.53%	0.20%	9.01%	0.00%	13.67%	**11.06%**
	**Average**		**34.48%**	**28.89%**	**9.28%**	**0.35%**	**11.93%**	**0.00%**	**17.31%**	
**Front vs Mid**	Volume	Shake	35.40%	37.31%	28.36%	49.74%	34.04%	48.48%	59.93%	**41.89%**
		Twist	35.68%	24.55%	32.97%	39.86%	24.96%	36.34%	46.46%	**34.40%**
		Bite	9.50%	14.73%	24.64%	17.47%	23.84%	23.56%	34.12%	**21.12%**
	Surface	Shake	34.98%	38.39%	27.77%	49.79%	33.18%	48.48%	60.25%	**41.83%**
		Twist	35.68%	24.33%	32.90%	39.81%	24.56%	36.34%	46.49%	**34.30%**
		Bite	10.12%	14.31%	25.01%	17.75%	24.46%	23.56%	34.26%	**21.35%**
	Length	Shake	38.63%	44.67%	29.16%	49.79%	33.58%	48.48%	65.68%	**44.28%**
		Twist	36.20%	24.19%	33.01%	39.87%	23.99%	36.34%	46.79%	**34.34%**
		Bite	6.15%	11.76%	24.21%	17.29%	25.34%	23.56%	36.41%	**20.67%**
	**Average**		**26.93%**	**26.03%**	**28.67%**	**35.71%**	**27.55%**	**36.13%**	**47.82%**	
**Front vs Back**	Volume	Shake	69.63%	67.16%	67.75%	70.34%	64.35%	76.17%	82.12%	**71.07%**
		Twist	68.69%	50.76%	66.70%	64.16%	58.33%	53.46%	67.29%	**61.34%**
		Bite	12.21%	16.10%	32.14%	25.47%	40.96%	32.76%	46.16%	**29.40%**
	Surface	Shake	69.39%	67.95%	67.47%	70.39%	63.02%	76.17%	82.27%	**70.95%**
		Twist	68.59%	50.64%	66.67%	64.21%	58.19%	53.46%	67.30%	**61.29%**
		Bite	13.30%	15.72%	33.49%	25.81%	42.14%	32.76%	46.35%	**29.94%**
	Length	Shake	71.90%	72.70%	68.20%	70.35%	62.42%	76.17%	84.56%	**72.33%**
		Twist	68.57%	49.72%	66.56%	64.13%	57.91%	53.46%	67.22%	**61.08%**
		Bite	7.48%	13.63%	32.21%	25.36%	43.82%	32.76%	49.82%	**29.30%**
	**Average**		**49.97%**	**44.93%**	**55.69%**	**53.36%**	**54.57%**	**54.13%**	**65.90%**	
**Mid vs Back**	Volume	Shake	53.00%	47.62%	54.97%	40.99%	45.87%	53.74%	55.37%	**50.22%**
		Twist	51.32%	34.76%	50.32%	40.42%	44.45%	26.89%	38.90%	**41.01%**
		Bite	3.02%	3.07%	9.94%	9.70%	22.38%	12.03%	18.29%	**11.21%**
	Surface	Shake	52.93%	47.99%	54.94%	41.03%	44.58%	53.74%	55.39%	**50.09%**
		Twist	51.16%	34.78%	50.33%	40.56%	44.56%	26.89%	38.89%	**41.03%**
		Bite	3.56%	3.09%	11.31%	9.81%	23.31%	12.03%	18.41%	**11.65%**
	Length	Shake	54.23%	50.66%	55.09%	40.95%	43.36%	53.74%	55.00%	**50.43%**
		Twist	50.74%	33.69%	50.09%	40.35%	44.61%	26.89%	38.39%	**40.68%**
		Bite	1.66%	3.10%	10.57%	9.77%	24.66%	12.03%	21.12%	**11.84%**
	**Average**		**35.74%**	**28.75%**	**38.62%**	**30.40%**	**37.53%**	**30.89%**	**37.75%**	

**Notes.**

Taxon abbreviations Ot
*Osteolaemus tetraspis*
Cm
*Crocodylus moreletii*
Cng
*Crocodylus novaeguineae*
Ci
*Crocodylus intermedius*
Cj
*Crocodylus johnstoni*
Mc
*Mecistops cataphractus*
Ts
*Tomistoma schlegelii*

**Figure 3:**
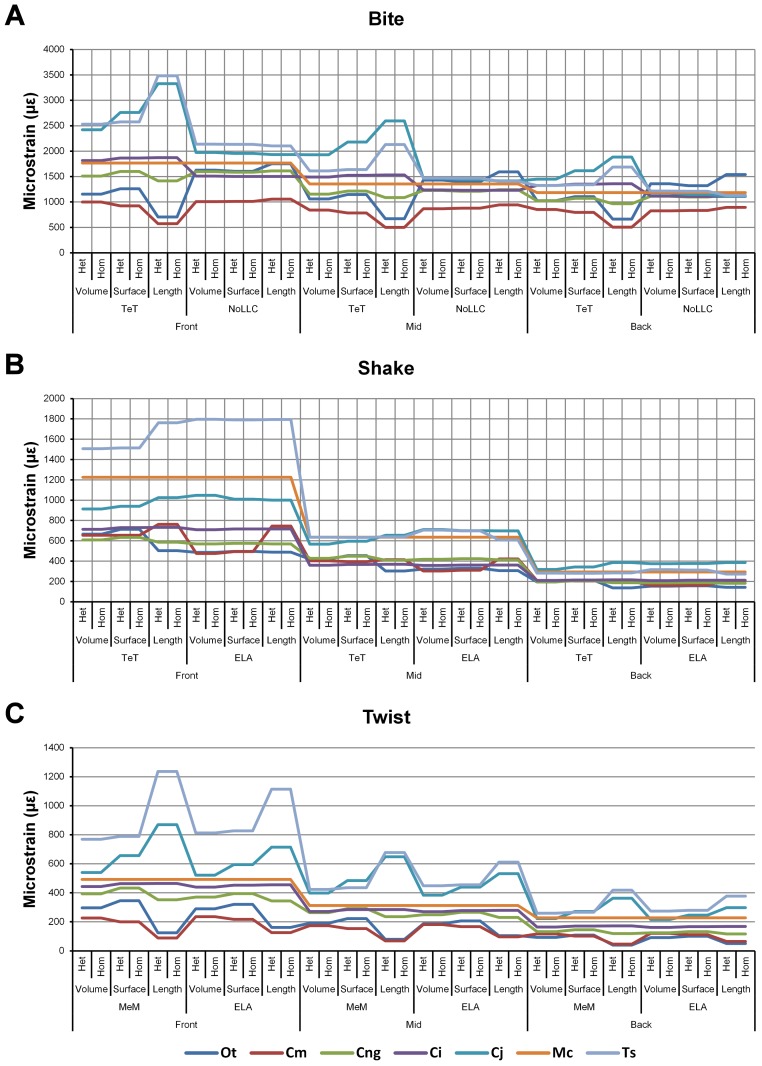
Pattern. The strain response of each species model for all *conditions* provides a graphical representation of quantitative *pattern* of results. *Conditions* are separated into biting (A), shaking (B), and twisting (C) feeding behaviours, and are subsequently labelled according to the combination of modelling factors used in that simulation. Front, Mid, and Back indicate simulations at front, mid and back bite positions respectively, while Surface, Volume, and Length indicate surface area, volume, and length scaling respectively. HET and HOM indicate simulations with isotropic heterogeneous and isotropic homogeneous material properties respectively, while TeT (‘tooth equals tooth’), NoLLC (‘no linear load case’), ELA (‘equal lever arm’), and MeM (‘moment equals moment’) each indicate the type of linear load case used in the simulation. Under biting, TeT simulates all species biting with identical ‘resultant’ bite force to *M. cataphractus*, while NoLLC simulates all species biting at their maximal muscle force. Under shaking, TeT simulates an identical magnitude of shake force to *M. cataphractus,* while ELA simulates shaking prey of identical mass at the same frequency. Under twisting, MeM simulates an identical magnitude of twisting force, while ELA simulates a constant ratio of skull width to twisting force between each species. Taxa are colour-coded. Taxon abbreviations: Ot, *Osteolaemus tetraspis*; Cm, *Crocodylus moreletii*; Cng, *Crocodylus novaeguineae*; Ci, *Crocodylus intermedius*; Cj, *Crocodylus johnstoni*; Mc, *Mecistops cataphractus*; Ts, *Tomistoma schlegelii*. Note that for shaking (B) feeding behaviours there is a much more pronounced reduction in microstrain (for all species models) when comparing a front to a mid bite position than comparing a mid to a back bite position. For twisting (C) feeding behaviour scaling to length results in the largest variation of microstrain; this is also true for biting (A) with the exception of *conditions* also including NoLLC, where there is little visible difference between scaling types.

**Figure 4:**
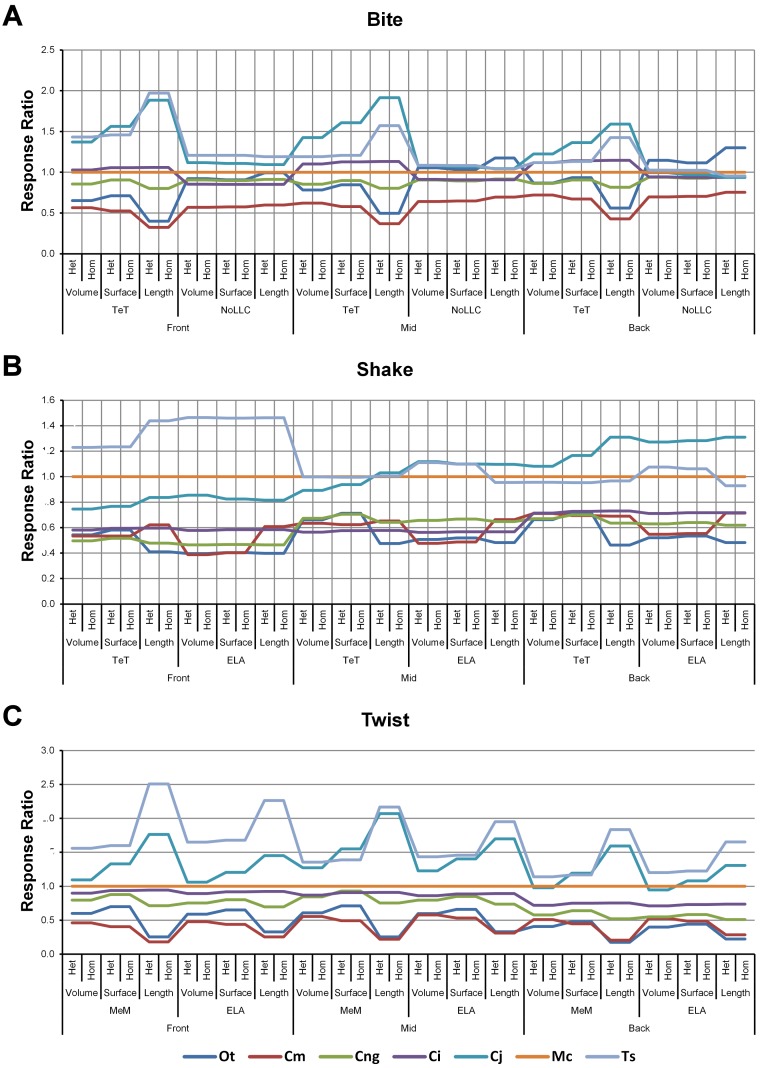
Standard pattern. The strain response of each species model is standardised to that of *M. cataphractus* for individual *conditions*, showing the relative performance of each species (or standard pattern). This ‘Response ratio’ is calculated as a ratio of the strain response in each species (ε_*sp*_) model (coded by colour) to strain in *M. cataphractus* (ε_*Mc*_) for individual *conditions*, i.e., ε_*sp*_/ε_*Mc*_. *Conditions* are separated into biting (A), shaking (B), and twisting (C) feeding behaviours, and are subsequently labelled according to the combination of modelling factors used in that simulation. Front, Mid, and Back indicate simulations at front, mid and back bite positions respectively, while Surface, Volume, and Length indicate surface area, volume, and length scaling respectively. HET and HOM indicate simulations with isotropic heterogeneous and isotropic homogeneous material properties respectively, while TeT (‘tooth equals tooth’), NoLLC (‘no linear load case’), ELA (‘equal lever arm’), and MeM (‘moment equals moment’) each indicate the type of linear load case used in the simulation. Under biting, TeT simulates all species biting with identical ‘resultant’ bite force to *M. cataphractus*, while NoLLC simulates all species biting at their maximal muscle force. Under shaking, TeT simulates an identical magnitude of shake force to *M. cataphractus,* while ELA simulates shaking prey of identical mass at the same frequency. Under twisting, MeM simulates an identical magnitude of twisting force, while ELA simulates a constant ratio of skull width to twisting force between each species. Taxon abbreviations: Ot, *Osteolaemus tetraspis*; Cm, *Crocodylus moreletii*; Cng, *Crocodylus novaeguineae*; Ci, *Crocodylus intermedius*; Cj, *Crocodylus johnstoni*; Mc, *Mecistops cataphractus*; Ts, *Tomistoma schlegelii*. Interestingly for twisting (C) feeding behaviours there is relatively little cross over between species model traces across all of the *conditions*, indicating that there is little change in the ranked order of species models between *conditions.* However, it’s important to note that the relative response (‘Response ratio’) of each species model shows considerable variation across *conditions*.

**Figure 5:**
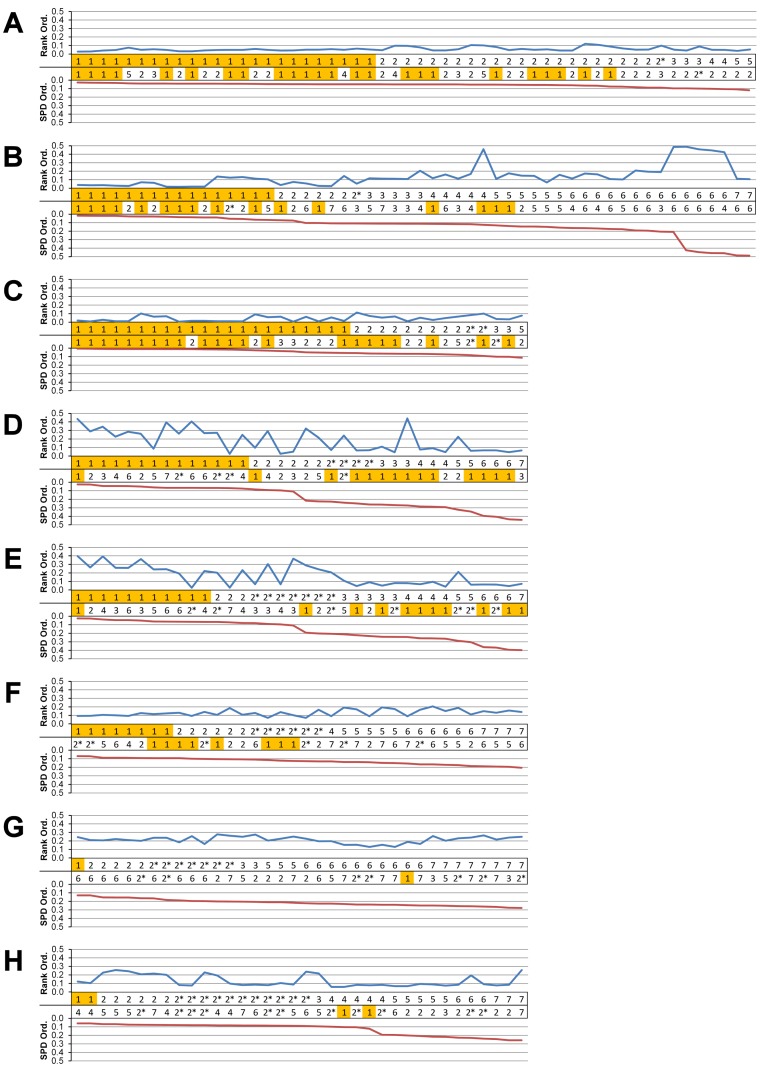
Standard pattern difference summary. Standard pattern difference (SPD) is the difference in values of standard pattern for individual species models between *condition pairs* within comparative *sets*, i.e., the difference in the relative performance of each species model to *M. cataphractus*. Between comparison *sets* (e.g. HET vs HOM) the average SPD of all species models is calculated for each *condition-pair* giving an overall measure of pattern difference. For each comparison *set* this average SPD for each *condition* is plotted two ways: (1) ‘Rank Order’ (above central horizontal line) orders *conditions* from best to worst (left to right) consistency in predictive rank (blue trace), where predictions of rank for each *condition* comparison are numbered according to whether 1, 2, 3, 4, 5, 6, or 7 species models had different ranks (1 indicates identical predictions - coloured orange); (2) ‘SPD Order’ orders *conditions* from lowest to highest (left to right) average SPD (red trace). High absolute values of either the red or blue traces indicate large (averaged across all species models) differences in standard pattern, i.e., large differences in relative performance. Ordering SPD in these two ways allows visualisation of the correlation between predictive rank and overall differences in the pattern of results. This figure summarises the broad trends in SPD. However, for greater details see the supplementary figures indicated in the following: (A) isotropic heterogeneous vs isotropic homogeneous material properties (Fig. S2), (B) Linear Load Case comparisons (Fig. S10), (C) volume- vs surface-scaling (Fig. S6), (D) volume- vs length-scaling (Fig. S7), (E) surface- vs length-scaling (Fig. S8), (F) front vs mid bite positions (Fig. S14), (G) front vs back bite positions (Fig. S15), (H) mid vs back bite positions (Fig. S16). Note that linear load case comparisons (B) and all bite position comparisons (F–H) have a bigger effect on the results than material properties (A) or volume vs surface area scaling (D).

**Figure 6:**
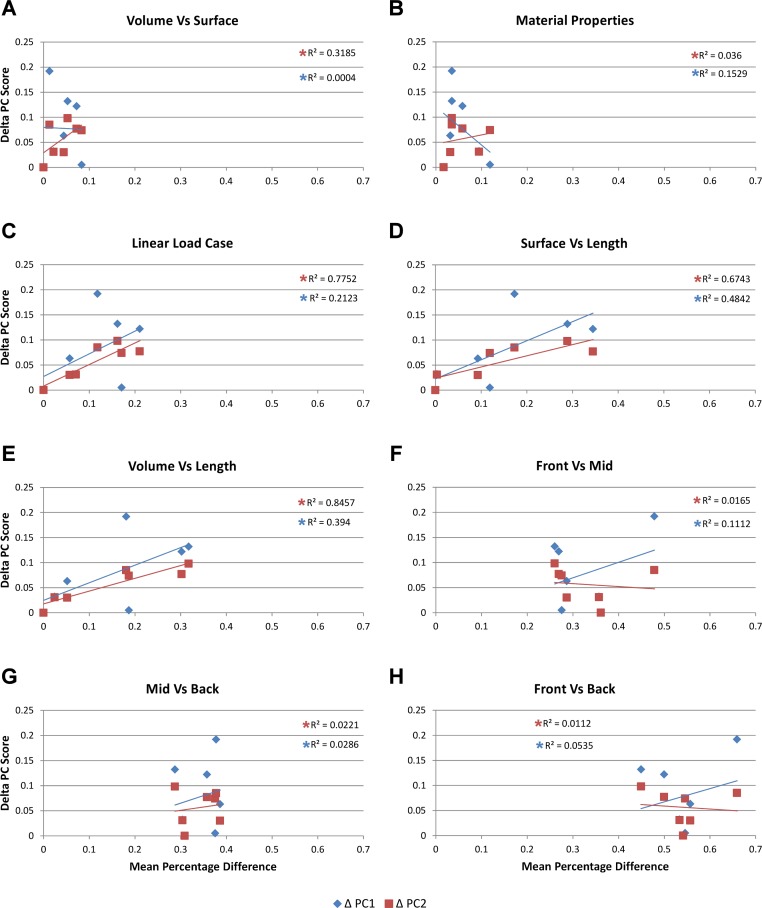
Mean percentage difference for each modelling factor vs PC scores. The relative difference in shape is calculated using principal component values (PC1 and PC2) from Walmsley et al. (2013) by taking the difference between all species models to that of *M. cataphractus* for PC1 and PC2, yielding a ΔPC1 and ΔPC2 value for each species model. These are plotted against the mean percentage difference values of each species for each comparison *set*. Note the good correlation with shape for Linear Load Cases, surface- vs length-scaling, and volume- vs length-scaling for ΔPC2 measures of shape. (A) Volume- vs surface-scaling, (B) isotropic heterogeneous vs isotropic homogeneous material properties, (C) TeT/MeM vs NoLLC/ELA Linear Load Cases, (D) surface- vs length-scaling, (E) volume- vs length-scaling, (F) front vs mid bite position, (G) mid vs back bite position, (H) front vs back bite position. Note that TeT (‘tooth equals tooth’), NoLLC (‘no linear load case’), ELA (‘equal lever arm’), and MeM (‘moment equals moment’) each indicate the type of linear load case used in the simulation. Under biting, TeT simulates all species biting with identical ‘resultant’ bite force to *M. cataphractus*, while NoLLC simulates all species biting at their maximal muscle force. Under shaking, TeT simulates an identical magnitude of shake force to *M. cataphractus,* while ELA simulates shaking prey of identical mass at the same frequency. Under twisting, MeM simulates an identical magnitude of twisting force, while ELA simulates a constant ratio of skull width to twisting force between each species.

Consistency in ranking (Table 3) was very high, with 24 of the 54 *condition* pairs predicting identical rankings, and a further 22 pairs differing in the rank of 2 models only. Of the remaining *condition* pairs, 5 differed in rankings by 3 or 4 species models, and there was 2 instances of ‘5 out’(for a detailed account of how well each *condition* pair predicted rank see Fig. S2). Charts of pattern (Fig. 3) and standard pattern (Fig. 4) likewise show that for each HET-HOM *condition* pair, strain values are very similar (horizontal parts of the trace). Standardised pattern difference (SPD) showed high consistency between *conditions* when ordered either by rank or mean standardised pattern difference (Fig. 5A), with low values of mean SPD (<0.1ε_*Mc*_) throughout. Mean percentage difference showed no correlation with shape as measured by ΔPC1 and ΔPC2 (Fig. 6B).

**Table 3:** Difference in predicted rank. The difference column classifies the type of difference observed in rankings. Labels are used as shorthand to indicate how well rank predictions correlate between input *conditions* (see Fig. 5, Figs. S2, S6–S8, S10 and S14–S16 for label implementation); low numbers indicate good correlation, while high indicates poor correlations. ‘2 out’ indicates that rankings differed only by inverting 2 species that were next to each other, while ‘3 out’ re-ordered 3 species that were next to each other, etc. ‘2 out*’ indicates a special case where two pairs of species are inverted at different ends of the ranking scale. Values in all other columns mark the number of occurrences observed for each pairwise comparison.

Difference	Labels	HET vs HOM	Vol vs Surf	Vol vs Len	Surf vs Len	TeT/MeM vs NoLLC/ELA	Front vs Mid	Front vs Back	Mid vs Back
Identical	1	24	22	14	11	16	8	1	2
2 out	2	22	9	6	3	6	6	5	6
2 out*	2*	1	2	4	7	1	6	7	11
3 out	3	3	2	3	5	5	0	2	1
4 out	4	2	0	3	4	5	1	0	5
5 out	5	2	1	2	2	6	5	3	5
6 out	6	0	0	3	3	13	6	10	3
7 out	7	0	0	1	1	2	4	8	3

### Scaling

**Figure 7:**
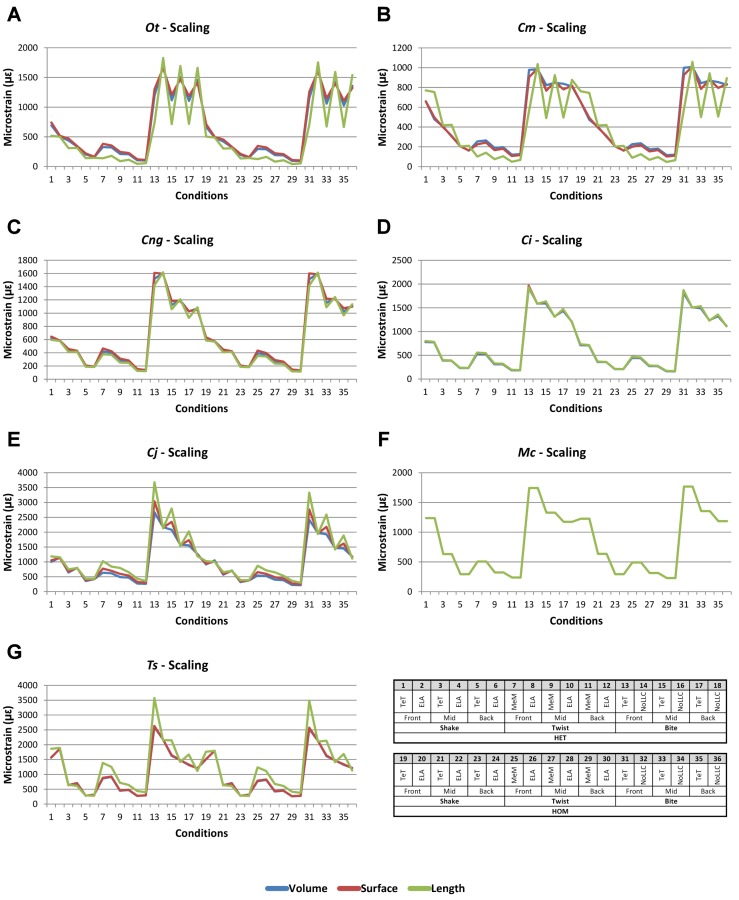
Scaling *signal* to simulation *conditions.* Simulation *conditions* are arbitrarily numbered from 1 to 36 (labelled bottom right). For each *condition* the response to scaling models to the same volume (blue), surface (red) and length (green) as *M. cataphractus* are graphed alongside each other. TeT (‘tooth equals tooth’), NoLLC (‘no linear load case’), ELA (‘equal lever arm’), and MeM (‘moment equals moment’) each indicate the type of linear load case used in the simulation. Under biting, TeT simulates all species biting with identical ‘resultant’ bite force to *M. cataphractus*, while NoLLC simulates all species biting at their maximal muscle force. Under shaking, TeT simulates an identical magnitude of shake force to *M. cataphractus,* while ELA simulates shaking prey of identical mass at the same frequency. Under twisting, MeM simulates an identical magnitude of twisting force, while ELA simulates a constant ratio of skull width to twisting force between each species. Note, in general, volume and surface scaling track closely to one another while length tends to show the greatest deviation. (A) Ot, *Osteolaemus tetraspis*, (B) Cm, *Crocodylus moreletii*, (C) Cng, *Crocodylus novaeguineae*, (D) Ci, *Crocodylus intermedius*, (E) Cj, *Crocodylus johnstoni*, (F) Mc, *Mecistops cataphractus*, (G) Ts, *Tomistoma schlegelii.*

Strain in volume-scaled models closely matched that of surface-scaled models across all *condition* pairs. The *signal* of models track closely (Fig. 7), and consistency in rankings is high (22 identically ranked *condition* pairs and 9 pairs that differ by two models, out of a total of 36 *condition* pairs in the *set*). Similarly, pattern (Fig. 3), standard pattern (Fig. 4), and standard pattern difference (Figs. 5C–5E), all show very small differences between volume- and surface-scaling.

For each of these qualitative and quantitative measures, length-scaled models exhibited quite different strain values from both volume- and surface-scaled models across all *condition* pairs. Rankings (Table 3) for length- vs volume-scaled models had 14 identical predictions, and 6 *conditions* that differed in the order of 2 species, out of a total of 36 conditions; for length- vs surface-scaled the equivalent numbers were 11 and 3 respectively. Plots of *signal* (Fig. 7) indicate that, while scaling to length follows the same broad trend as volume- and surface-scaling, for individual *conditions* it consistently shows the greatest deviation (akin to noise) in its *signal*.

This pattern of results is also evident in percentage difference values; volume- vs surface-scaled models show the smallest differences in strain response overall, with the average for individual species ranging from ≈1% for *T. schlegelii*, through to ≈8% for *C. johnstoni* (Table 2 and Fig. S3). Volume- vs length-scaling shows much larger averages for individual species, from ≈2% for *C. intermedius*, through to ≈32% for *C. moreletii* (Table 2 and Fig.S4). Similarly surface- and length-scaling show large differences, from <1% for *C. intermedius*, through to ≈34% for *O. tetraspis* (Table 2 and Fig. S5). *Crocodylus intermedius* shows very little difference between all three scaling parameters, with a maximum average difference of ≈2%, and absolute max difference of ≈5% for volume- and length-scaled simulations (Table 2 and Fig. S6). Between species models the largest and smallest differences were identical for volume- and length-scaling, and surface- and length-scaling, with *C. intermedius* and *C. novaeguineae* displaying the smallest differences, and *O. tetraspis* and *C. moreletii* displaying the largest (Table 2, Figs. S4 and S5). Between volume- and surface-scaling *C. intermedius* and *T. schlegelii* show the smallest differences, while *C. johnstoni* and *O. tetraspis* show the largest (Table 2 and Fig. S3).

Between *conditions*, for all species models and each scaling comparison, the greatest differences were for those that involved twisting, although this was less pronounced in *C. intermedius* between surface- and length-scaling (Table 2, Figs. S3–S5). These large differences are also apparent in pattern (Fig. 3), and standard pattern (Fig. 4), which both show larger variation between scaling parameters for twist when compared to either bite or shake. Additionally, the largest differences for SPD are overwhelmingly dominated by twist feeding behaviours, which all fall in the worst half of SPD, and those *conditions* also involving MeM linear load cases consistently perform worst of all (Figs. S6B, S7B and S8B).

Qualitative and quantitative measures of *sets* gave inconsistent results for comparisons between length- and either volume- or surface-scaled models, in that those *conditions* that predict identical rank show some of the largest differences in SPD (Figs. 5D–5E, Figs. S7 and S8). Comparing between volume- and surface-scaled models shows much higher consistency between qualitative and quantitative measures; conditions with the smallest variation in SPD were predominantly identical or near predictions of rank (Fig. 5C and Fig. S6).

Mean percentage differences between volume- and surface-scaled models show no correlation with shape, as measured by ΔPC1 and ΔPC2; however, the larger variation in results between length- and both volume- and surface-scaled models showed some correlation with shape (Figs. 6A, 6D and 6E). In both cases mean percentage difference correlated well with ΔPC2, and poorly with ΔPC1, with surface-length comparisons showing *r*^2^ values of 0.67 and 0.48, and volume-length 0.85 and 0.39, for ΔPC2 and ΔPC1 respectively.

### Linear load cases

**Figure 8:**
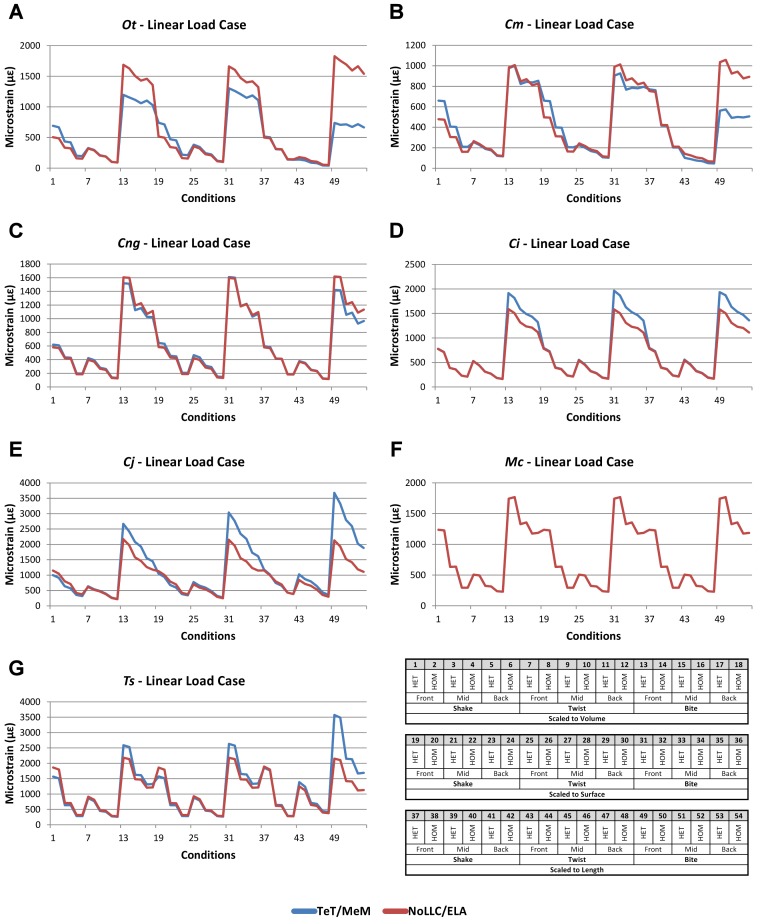
Linear Load Case *signal* to simulation *conditions.* Simulation *conditions* are arbitrarily numbered from 1 to 54 (labelled bottom right), for each *condition* the response to each Linear Load Cases (LLCs) is graphed alongside one another. TeT (‘tooth equals tooth’), NoLLC (‘no linear load case’), ELA (‘equal lever arm’), and MeM (‘moment equals moment’) each indicate the type of linear load case used in the simulation. Under biting, TeT simulates all species biting with identical ‘resultant’ bite force to *M. cataphractus*, while NoLLC simulates all species biting at their maximal muscle force. Under shaking, TeT simulates an identical magnitude of shake force to *M. cataphractus*, while ELA simulates shaking prey of identical mass at the same frequency. Under twisting, MeM simulates an identical magnitude of twisting force, while ELA simulates a constant ratio of skull width to twisting force between each species. Note that large difference between LLCs tends to occur at regular intervals corresponding to biting feeding behaviours. (A) Ot, *Osteolaemus tetraspis*, (B) Cm, *Crocodylus moreletii*, (C) Cng, *Crocodylus novaeguineae*, (D) Ci, *Crocodylus intermedius*, (E) Cj, *Crocodylus johnstoni*, (F) Mc, *Mecistops cataphractus*, (G) Ts, *Tomistoma schlegelii*.

Results between LLC models show large variation across *conditions*, both qualitatively and quantitatively. For some combinations of species model and *conditions*, TeT/MeM results correlate well with NoLLC/ELA, whilst for others the agreement is low. *Conditions* involving both volume-scaling and twisting show good correlations across all species, while *conditions* involving length scaling and biting show consistently poorer correlations, ranging from an average percentage difference of 13% for *C. novaeguineae* through to 58% for *O. tetraspis* (Table 2 and Fig. S9); *signal* also shows large differences for those *conditions* (Fig. 8). The largest deviations in SPD were always biting *conditions*, with the very worst also involving length scaling (Fig. S9B).

With respect to species models, *C. novaeguineae* shows good correlations, while *O. tetraspis* shows poor correlations overall, but even this is inconsistent; the *signal* for TeT/MeM models tracks NoLLC/ELA closely for volume-scaled twist conditions, but tracks poorly for length-scaled biting (Fig. 8A). Percentage difference between each pair of *conditions* shows considerable variation (Fig. S9), ranging from a minimum of 2% through to maximum of 59% for *O. tetraspis*. The largest mean percentage differences were for *O. tetraspis* (avg. ≈21%), and *C. johnstoni* (avg. ≈17%), with *C. novaeguineae* showing the smallest ≈6% (note that the *M. cataphractus* models have zero differences since LLCs are equal for this species model) (Table 2, Fig. S9).

Pattern (Fig. 3) and standardised pattern (Fig. 4) show small and large differences between LLCs; for example, in the *C. intermedius* models, shake and twisting *conditions* show small differences between LLCs, while large differences are seen for biting *conditions*. This variation in quantitative pattern is illustrated by the SPD (Fig. 5B), where mean standard pattern difference varies from almost indistinguishable through to ≈ 0.5ε_*Mc*_. Additionally SPD for individual species models ranged from almost indistinguishable from *M. cataphractus*, to more than 0.8 of that benchmark (Fig. S10).

Consistency in ranking (Table 3) was low, with only 16 of the 54 *condition* pairs predicting identical rankings, and a further 6 pairs differing in the rank of 2 models only. Of the remaining *conditions*, 10 were out by 3 or 4, and 21 reported substantially different rankings (out by 6 or 7). With respect to consistency between qualitative and quantitative results, predictive rank displayed an appreciable spread when ordered by SPD, but the smallest differences in pattern are still dominated by identical or near (‘2 out’) predictions of ranked order (Fig. 5B and Fig. S10); conditions that were qualitatively consistent were also quantitatively similar.

High variation in LLC results showed some correlation with shape; mean percentage difference showed good correlation with ΔPC2 (*r*^2^ = 0.77), but poor correlation with ΔPC1 (*r*^2^ = 0.21). This suggests that sensitivity of models to LLC is related to shape (Fig. 6C), particularly those aspects of shape captured within PC2 – inter-rami angle, followed closely by symphyseal length and mandibular width; see Figure 19 in Walmsley et al. (2013). Plots of *signal* support this observation, in that where differences are large, TeT/MeM over-predicts compared to NoLLC/ELA in species models with long and narrow rostra (Figs. 8D, 8E and 8G), and under predicts for those with more robust, broad and short rostra (Figs. 8A and 8B).

### Bite position

**Figure 9:**
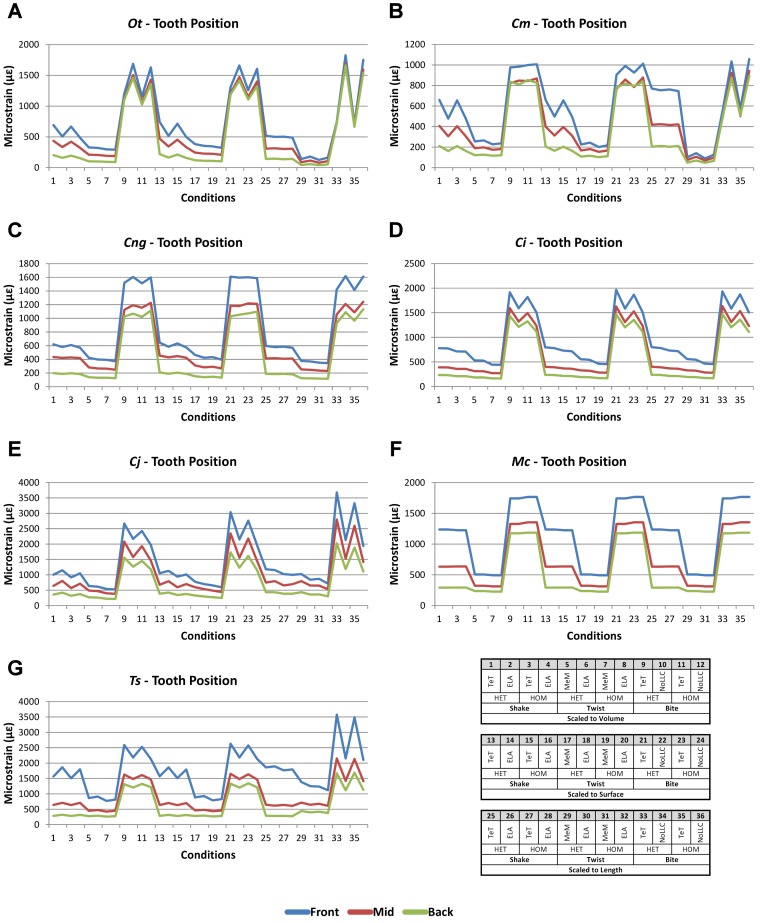
Bite position *signal* to simulation *conditions.* Simulation *conditions* are arbitrarily numbered from 1 to 36 (labelled bottom right). For each *condition* the response to simulating loads at front (blue), mid (red) and back (green) bite positions are graphed alongside each other. TeT (‘tooth equals tooth’), NoLLC (‘no linear load case’), ELA (‘equal lever arm’), and MeM (‘moment equals moment’) each indicate the type of linear load case used in the simulation. Under biting, TeT simulates all species biting with identical ‘resultant’ bite force to *M. cataphractus*, while NoLLC simulates all species biting at their maximal muscle force. Under shaking, TeT simulates an identical magnitude of shake force to *M. cataphractus,* while ELA simulates shaking prey of identical mass at the same frequency. Under twisting, MeM simulates an identical magnitude of twisting force, while ELA simulates a constant ratio of skull width to twisting force between each species. Note that despite differences in amplitude the general waveform of *signal* for front, mid, and back bite positions is consistent across all *conditions*. (A) Ot, *Osteolaemus tetraspis*, (B) Cm, *Crocodylus moreletii*, (C) Cng, *Crocodylus novaeguineae*, (D) Ci, *Crocodylus intermedius*, (E) Cj, *Crocodylus johnstoni*, (F) Mc, *Mecistops cataphractus*, (G) Ts, *Tomistoma schlegelii.*

Results between front, mid, and back bite positions exhibit appreciably large differences across all *conditions*, both qualitatively and quantitatively. All three bite positions show poor correlation in *signal* (Fig. 9), poor predictive rank (Table 3), large percentage differences (Table 2, Figs. S11–S13), as well as large differences in pattern (Fig. 3), standard pattern (Fig. 4), and standard pattern difference (Figs. 5F–5H and Figs. S14–S16).

The overall waveform of *signal* for front, mid, and back bite positions remains reasonably consistent across all *conditions* for each species, mainly varying in amplitude (Fig. 9); however, this variation is large (e.g., *M. cataphractus*) and not uniform throughout *conditions* (e.g., *O. tetraspis* shows smaller variation in bite *conditions* than in shake).

Pattern (Fig. 3) shows large differences between all three bite positions, where response decreases and compresses across all species models when moving from front to back positions. Although somewhat less noticeable, standard pattern (Fig. 4) also shows reasonable differences for all species models. SPD also shows large differences, with individual species models extending beyond 0.4 of *M. cataphractus* for most *conditions* (Figs. S14–S16), and averaging >0.1 of *M. cataphractus* across most *conditions* for front and mid, and front and back condition pairs (Figs. 5F–5G).

While percentage differences are typically large between all bite positions, mid and back show the smallest overall, with the average ranging from 29% for *C. moreletii* to 39% for *C. novaeguineae* (Table 2 and Fig. S13). Front and back show the largest difference, ranging from 45% for *C. moreletii*, through to 66% for *T. schlegelii* (Table 2 and Fig. S12), and similarly for front and mid, the average ranges from 26% for *C. moreletii*, through to 48% for *T. schlegelii* (Table 2 and Fig. S11).

Ranked order of specimen is highly sensitive to bite position, with a large proportion of simulation *conditions* resulting in substantially different predictions (Table 3). Of the 36 possibilities 15 were out by 5 or more between front and mid *condition* pairs, 21 for front and back, and 11 for mid and back. While identical predictions were only observed 8 times for front and mid bite position pairs, once for front and back, and twice for mid and back, slight differences were somewhat more frequent, particularly between front and back, and mid and back *condition* pairs (Table 3).

Comparisons between either front and back or mid and back bite *condition* pairs show a low consistency between qualitative and quantitative results, in that those *conditions* that predict identical rank show large differences in SPD, and the smallest differences in SPD are consistently very poor predictions of rank (Figs. 5G–5H and Figs. S15–S16). Between front and mid *condition* pairs, good predictors of rank spread appreciably when ordering *conditions* by SPD, although the best half of *conditions* ordered by SPD predominately consists of good predictors of rank (Fig. 5F and Fig. S14).

Between *conditions*, for all species models and each bite position comparison, the largest differences were for those that involved shaking – with the exception of *C. novaeguineae* between front and mid positions, whose largest differences were for twist *conditions* (Table 2, Figs. S11–S13). These large differences are also apparent in pattern (Fig. 3), and standard pattern (Fig. 4), where much larger variation is apparent between bite positions for shake compared to either bite or twist. The smallest SPD is also dominated by *conditions* involving biting, although somewhat less pronounced between mid and back positions; while the largest are dominated by *conditions* involving twisting, specifically those also involving HET material properties, which is less pronounced for front and mid positions (Figs. S14–S16).

Mean percentage differences show no correlation with shape, as measured by ΔPC1 and ΔPC2, for all three bite position comparisons (Figs. 6F–6H).

## Interpretation

### Material properties (Isotropic HET vs Isotropic HOM)

Qualitatively and quantitatively the selection of either HET or HOM material properties (as we calculated these) made little difference in the interpretation of results. This is evident from the small differences in *signal* (Fig. 2), percentage difference (Table 2 and Fig. S1), pattern (Fig. 3), standard pattern (Fig. 4), and standard pattern difference (Fig. 5A and Fig. S2), as well as the large proportion (46 of 54) of *conditions* that predict identical or near (2 out) specimen rankings (Table 3). Interestingly these differences are small despite HOM material properties for all species models being calculated from the average of *M. cataphractus*, and not from their own HET average (Table 4).

**Table 4:** Mass-conserved homogeneous material properties. Material properties used for all homogeneous models were that of *M. cataphractus*, and others are displayed here only for comparison. Note that the units used for density here is in tonnes per cubic millimetre (T/mm^3^).

Taxon	Density (T/mm^3^)	Young’s modulus (MPa)
*Osteolaemus tetraspis*	1.47E−09	12038
*Crocodylus moreletii*	1.54E−09	12958
*Crocodylus novaeguineae*	1.56E−09	13191
*Crocodylus intermedius*	1.49E−09	12313
*Crocodylus johnstoni*	1.49E−09	12292
*Mecistops cataphractus*	1.58E−09	13471
*Tomistoma schlegelii*	1.56E−09	13119

The fact that *conditions* involving twisting displayed the greatest sensitivity to the selection of material properties may relate to differences in material stiffness at the outer surface of HET models compared with HOM models; during elastic torsional loading material furthest from the axis of rotation carries a higher proportion of the load (Spotts & Shoup, 2004). This result suggests that torsional loads may be at least as important as bending loads in determining the distribution of cortical bone within beam-shaped skeletal elements.

### Scaling

Qualitatively and quantitatively, scaling to either surface or volume made little practical difference upon the results or their interpretation. This is evident from the small differences in *signal* (Fig. 7), percentage difference (Table 2 and Fig. S3), pattern (Fig. 3), standard pattern (Fig. 4), and standard pattern difference (Fig. 5C and Fig. S6), as well as the large proportion (31 of 36) of *conditions* that predict identical or near (2 out) specimen rankings (Table 3).

Comparing length- to either volume- or surface-scaling made a bigger difference in the results, displaying large differences in *signal* (Fig. 7), percentage difference (Figs. S4 and S5), all measures of pattern (Figs. 3, 4, 5D, 5E and Figs. S7–S8), and a large proportion of inconsistent rank predictions (Table 3). The higher sensitivity to length-scaling is related to the spectrum of skull shape in crocodilians, ranging from longirostrine through to brevirostrine taxa (Busbey, 1995; Langston, 1973; McHenry et al., 2006). Scaling to length is arguably appropriate for exploring the consequences of different head length morphologies and symphyseal morphologies, however this needs to be used very carefully; a brevirostrine animal with the same head length as a longirostrine would be a much larger animal with a much stronger skull. Differences between length- and either volume- or surface-scaling appear to be a function of shape, where the largest differences are seen in both relatively shorter and broader (*O. tetraspis* and *C. moreletii*) or longer and narrower (*T. schlegelii*), skulls than *M. cataphractus* (Table 2 and Fig. 7); additionally this is supported by the strong correlations with ΔPC2 scores (Fig. 6).

**Figure 10:**
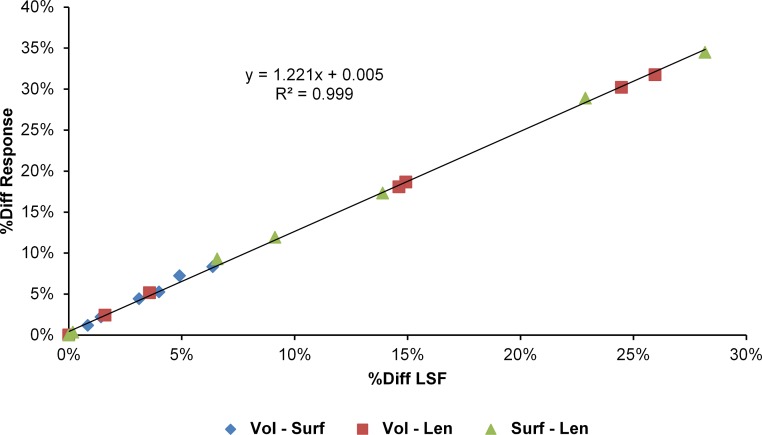
Difference in linear scaling factor vs difference in response for scaling parameters. The percentage difference between the Linear Scaling Factors (%Diff LSF) used to scale each species model to the same volume, surface, or length as *M. cataphractus* is plotted against the average percentage difference between the responses of each species model (%Diff Response) at each re-scaled size. Note the strong linear relationship between the differences in LSF and differences in response.

The differences in results between all three scaling parameters are a function of the proportional difference between the linear scaling factors (LSF) used to scale models to volume, surface, and length (Fig. 10). Larger proportional differences between LSFs directly translate to larger differences in the response of models after scaling to one parameter or another. This explains why length-scaled models have such different results to both volume- and surface-scaled models; the difference between the LSFs of length- compared to both volume- and surface-scaling is proportionally larger than between volume- and surface-scaling.

Similar to material properties, *conditions* involving twisting display the greatest sensitivity to the selection of scaling parameters, consistently showing the largest absolute percentage difference across all species (Table 2, Figs. S3–S5), and dominating the largest standard pattern difference (Figs. S6B, S7B and S8B), particularly those *conditions* also involving MeM Linear Load Cases. In this regard conclusions relating to twisting feeding behaviours should be considered carefully, since the selection of one scaling parameter over another has a substantial influence over how the results would be interpreted.

Results show a high sensitivity both qualitatively and quantitatively to simulations where models are scaled to length as opposed to either surface or volume, and while this doesn’t speak to the appropriateness of one scaling parameter over another, it does suggest that the selection of length as a scaling technique should be well justified, since it is likely to dramatically change the pattern of results and their interpretation.

### Linear load cases

Selection of appropriate LLC is important, and the interpretation of results would be largely dependent on which were used in the simulations. This is evident from the large difference in *signal* for most species across a number of simulation *conditions* (Fig. 8), the high proportion (26 of 54) of *conditions* that badly (4 or more out) predict rankings (Table 3), and large SPD – averaging > 0.1ε_*Mc*_ for most simulation *conditions* (Fig. 5B). Qualitatively *conditions* that involve biting show the greatest sensitivity to the selection of LLCs, showing very poor predictions of ranked order in addition to accounting for all of the largest differences in SPD (Fig. S10B).

In this analysis most *conditions* that show good predictions of rank also show the smallest variation in SPD (Fig. 5B and Fig. S10B). This means that those *conditions* that show good predictions of rank are also quite similar in regards to their pattern of results, and thus selection between the LLCs presented here becomes somewhat arbitrary since each yield similar results. However, this information could only be acquired through an extensive sensitivity analysis such as this, and is unlikely to remain true for other comparative datasets.

Quantitatively, absolute percentage differences (Fig. S9) vary considerably with respect to scaling parameter, feeding behaviour, and the model species, displaying both very large and very small difference for different combinations of these parameters. The only distinctive trend is that the largest difference for all species occurring under *conditions* combining biting and length-scaling. This range of difference in the results suggests that LLCs are far more sensitive to combinations of factors than to any one factor, particularly those combinations relating to the variation in skull shape which changes the values used for each LLC. In the example of shaking and the two LLCs used here, one simulates an identical lateral force across all species, and the other simulates a constant ratio of outlever-length to lateral force – i.e., shaking identical mass at an identical frequency. For the applied force for each of these simulations to be identical (and thus the microstrain results), scaling must be such that outlever-length is identical for each model. In this way the small differences between LLCs for shaking manifest as a result of outlever-length being very close to that of *M. cataphractus* at the rescaled size, and is most evident in *conditions* involving length-scaled shaking where differences in out-lever length are smaller for all species (Fig. S9).

In many comparative analyses an arbitrarily selected (normally equal) load is simulated on all specimens, with the prevailing logic that after size is accounted for, all that remains to influence biomechanical response is shape. Importantly, by simulating identical forces across all species, information about the functional aspect of that feeding behaviour is lost. In shaking, simulation of an equal lateral force results in each animal NOT shaking a prey item of the same mass at an identical frequency. Conversely by simulating identical mass and frequency, the selection of an appropriate scaling parameter becomes much more important since the simulated force is calculated by outlever-length, which is an aspect of shape determined by the scaling parameter. Similarly the forces calculated for twisting and biting would also be influenced by aspects of shape that can be over- or under-stated as a result of scaling parameter selection.

### Bite position

Qualitatively, selection of either front, mid, or back bite positions in simulations is important, and interpretation of results would be largely dependent on which were used. This is evident from the large differences in *signal* between all three bite positions (Fig. 9), the small proportion (8, 1, and 2 of 36 for front-mid, front–back, and mid-back comparisons respectively) of *conditions* that predict identical rankings (Table 3), and the large differences in SPD, averaging >0.1ε_*Mc*_ for most simulation *conditions* (Figs. 5F–5H). From a quantitative point of view, those *conditions* involving bite show the least sensitivity to the selection of bite position across all species, consistently showing the smallest absolute percentage differences (Figs. S11–S13), and additionally dominating the smallest differences in SPD for all comparisons (Figs. S14–S16). However, absolute percentage difference and SPD for bite *conditions* is much larger than that seen for either material properties (Figs. S1 and S2) of volume- vs surface-scaling (Figs. S3 and S6).

The combination of large differences in pattern (Fig. 3), standard pattern (Fig. 4), standard pattern difference (Fig. 5), the small number of identical rank predictions (Table 3), the large absolute percentage differences (Table 2), and the large differences in *signal* (Fig. 9) between all three bite positions, together illustrates a very important point for comparative biologists. Broad assertions (or interpretations) about skull optimisation for a specific feeding type cannot be inferred from simulations at a single bite position, since the pattern of results between specimen changes dramatically depending on the selection of bite position. For example some skulls may be better optimised for back, or mid biting, than they are for front biting, so simulations of front biting should only be used to make interpretations about that specific behaviour and not extended to biting in general. For a more comprehensive understanding of skull optimization for a specific feeding behaviour when multiple bite positions are feasible, each bite position must be analysed separately and conclusions drawn from the aggregation of all data. Further to this point, where observational data relating to feeding behaviours is available, it should be incorporated into the simulations so that comparisons remain logical in the context of their biological reality. If species A, B, and C are all know to engage in shake feeding behaviours but species B tends to grip prey at its mid bite position, while species A, and C tend to grip prey at a front bite position, the most logical comparison is not simulating all 3 shaking at a front bite position, but A and C at front, and B at mid. Simulations performed in this way, guided heavily by accurate observational data, are likely to better reflect biological reality, and additionally increase confidence in the results they provide.

### Feeding behaviour

For comparative simulations conclusions can only be drawn for the specific feeding behaviour being compared, and blanket conclusions relating to performance cannot be inferred from one behaviour to another. For instance if the results from a simulation relating to biting suggests one specimen performs better than another, this does not mean that for a different feeding behaviour (i.e., twisting or shaking) the same relationship exists. While simulations relating to different feeding behaviour are used here, we do not compare predictions about overall skull performance between different feeding behaviours, since they are functionally incomparable.

### Overall patterns

**Figure 11:**
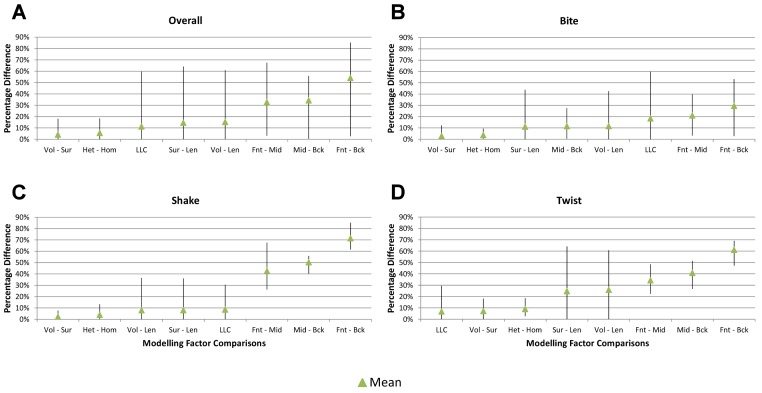
Min, Max, and Mean percentage differences. The range of percentage differences for each modelling factor comparison is indicated by the upper (maximum % difference) and lower (minimum % difference) extent of vertical bars, ordered left to right based on their aggregated average. Overall (A) includes differences from all feeding behaviours, while bite (B), shake (C), and twist (D) only include differences from their respective feeding types. Note that the order of modelling factor comparisons changes between biting (B), shaking (C), and twisting (D), suggesting that different feeding types are more (or less) sensitive to different modelling factors.

Model sensitivity varied between modelling factors; the highest sensitivity was for bite position with an average percentage difference >30% for all bite position comparisons (Fig. 11A). All other modelling factor comparisons averaged <20%, with volume- vs surface-scaling, and material property selection showing the smallest differences, both averaging ≈5%. Individual feeding behaviours show varied degrees of sensitivity to modelling factors, with bite being the least sensitive, averaging <30% for all modelling factors (Fig. 11B). Linear Load Case comparisons are not directly comparable between feeding behaviours (since each scale loads differently — see methods); however, bite load cases were highly sensitive to LLC, far more so than either shake or twist. This is likely due to the functional difference between the TeT and NoLLC *conditions*; TeT simulates a standardised bite force across all species models, so that all but *M. cataphractus* are biting either above or below their calculated maximal bite force, while NoLLC simulates maximal muscle recruitment for each species model. Shake shows the highest sensitivity to all bite position comparisons (average >40%) compared to either bite or twist, and very low sensitivity (average <10%) to all other modelling factors (Fig. 11C); the high sensitivity to bite position is likely a result of the applied loads being a function of outlever-length, which changes dramatically between bite positions. While less sensitive to bite position than shake, twist also shows high sensitivity to scaling, specifically to length- vs either volume- or surface-scaling (Fig. 11D).

Recent comparative analyses have directed a lot of attention to the importance of scaling to either volume or surface (Dumont, Grosse & Slater, 2009) in addition to the need for accurate material properties (Wroe et al., 2007b). However, our results show that our models are not nearly as sensitive to these factors as they are to bite position or linear load cases (at least, for the way we have modelled these). Interestingly, these are generally accepted in the literature without question. In particular, bite position has a large influence on the pattern of results, and the high sensitivity of models to bite position emphasises the importance of using empirical data on behaviour as input variables for any comparative modelling analyses; specifically, that it’s much more important than the validation of material properties of bone or the selection of either volume- or surface-scaling.

## Discussion

In many of the previous validation and sensitivity studies, material properties are often found to have significant influence on FEA results (Bright & Rayfield, 2011b; Cox et al., 2011; Porro et al., 2011; Reed et al., 2011; Strait et al., 2005). That these findings differ from those in our study likely arises as a result of study design: (1) we do not consider the specific location of strain response in our models, unlike other analyses (Bright & Rayfield, 2011b; Strait et al., 2005); (2) we compare between two methods of applying material properties that result in the same bulk density, in contrast to varying material properties across a range of values (Cox et al., 2011); and (3) our simulation does not make any comparisons to either orthotropic (Reed et al., 2011; Strait et al., 2005) or anisotropic (Porro et al., 2011) material properties. Of these, the second may be the most important; we emphasise that the bulk properties of materials for isotropic homogeneous models was chosen based on the properties given to that of an isotropic heterogeneous one. While this study does not speak to the accuracy (in terms of matching reality) of either method used here for applying material properties, it does suggest that on a broad scale (i.e., across multiple taxa) the selection of either method would have little influence on both the absolute and interspecific pattern of results (regardless of the combined variance of other modelling factors assessed here). This is an important result for comparative biologists confronted with this specific modelling decision, as applying isotropic heterogeneous properties in this way can be time-consuming, and in the case of analyses incorporating fossil taxa may be unfeasible.

Our simulations show that of all the modelling factors assessed, bite position was found to have the most significant influence over the results, and it should be noted that studies by Fitton et al. (2012) and Cox et al. (2011) have also found that bite position had significant influence over the results. This is an important result to consider for comparative studies, as it emphasises that simulations are particularly sensitive to the functional context of the feeding behaviour being simulated.

As with all sensitivity studies, we note that there is no way of gauging the extent to which these models are actually matching reality without detailed validation studies. Additionally, results here may not be directly applicable to other comparative datasets, and thus inferences on other datasets should be made with caution. This aside, we present multiple techniques for investigating differences in comparative studies, which together provide a framework for assessing sensitivity to specific modelling factors. Where models are shown to be sensitive to the values chosen for those modelling factors, those input values should be based upon empirical data; even in the absence of validation, this will increase the chances of the model producing relevant results. While the particular results presented are specific to the modelling factors and the species simulated here, our study does provide insight into which factors in a broad scale comparative FEA have the most effect on results and interpretation. For the crocodilian models analysed, broad-scale biological factors such as behaviour, relative head length, and bite position have much greater effect on comparative outcomes than technical factors such as material property regime and volume vs. surface scale correction.

## Conclusions

As computational modelling techniques evolve from being a novel approach through to being common practice, it is important to assess the reliability of models that are used within the field of comparative biomechanics. Consideration of the complex interactions between modelling factors, and the extent to which they influence the results, is an essential step where high levels of confidence in results are required; this relies upon confidence in both the selection of modelling factors and their associated input values. The preferred method for assessing model reliability is validation, but where validation is not possible or is logistically difficult, sensitivity analyses can be used to identify which modelling factors have a large influence over results. Identifying those factors allows their input values to be determined from empirical data, rather than an assumed value.

In the context of different feeding behaviours, sensitivity analyses should not be inferred between feeding behaviours as the relative influence of individual modelling factors varies between different behaviours. Since differences are proportional to shape in some cases, modelling factor values used for one comparative dataset may not be appropriate for another, as the differences in shape may be more (or less) sensitive to identical modelling factors. Overall, the accuracy of input data is paramount when performing comparative analyses, and biological context should be taken into account, particularly in regards to feeding behaviours at different bite positions.

Ultimately, it is important not to treat FEA as a black box, where reasonable assumptions are automatically assumed to only have small influences on the pattern of results. There is no ‘silver bullet’ procedure to ensure the accuracy of results, and for each comparative dataset some modelling factors will be more (or less) valid for a specific question, so results and assumptions should be scrutinised rigorously before making any broad scale conclusions. Caveats aside, the feeding behaviours (and bite positions) tested here had by far the biggest influence on the results, i.e., the biological hypothesis related to the examined behaviour has the biggest influence on comparative results. This is encouraging, because it suggests that FEA’s ability to resolve comparative signals, and therefore test biological hypotheses, overcomes the noise of uncertainty within parameter space. Biological factors such as morphology, function, behaviour, and natural history are the starting points for hypotheses testable with FEA, and endpoints of comparative inference.

## Supplemental Information

10.7717/peerj.204/supp-1 Figure S1 Percentage difference between heterogeneous and homogeneous material properties Absolute percentage difference between models simulated with HET and HOM material properties. Columns are individually colour coded according to the highest and lowest differences for that species using the inbuilt conditional formatting function in Excel. Hot colours (red and orange) indicate large differences (the largest in red) while cooler colours (green and yellow) indicate smaller differences (the smallest in green). Note that the largest differences consistently occur under twisting load cases. Taxon abbreviations: Ot, *Osteolaemus tetraspis*; Cm, *Crocodylus moreletii*; Cng, *Crocodylus novaeguineae*; Ci, *Crocodylus intermedius*; Cj, *Crocodylus johnstoni*; Mc, *Mecistops cataphractus*; Ts,*Tomistoma schlegelii*.

10.7717/peerj.204/supp-2 Figure S2 Standard Pattern Difference between heterogeneous and homogeneous simulations Response is standardised for each species with respect to *M. cataphractus* for HET and HOM simulation *conditions*, and the difference is then plotted for each *condition*. For an individual species a difference of zero indicates that it performs exactly the same (relative to *M. cataphractus*) for HET and HOM material properties under that *condition*; and conversely, large deviations from zero indicate large differences in relative performance. (A) Orders *conditions* (left to right) by consistency in rank predictions, and (B) orders *conditions* (left to right) from the smallest average SPD through to the largest. For each *condition*, comparisons between ranked order is indicated by numbers, where ‘1’ (also marked by red stars) indicates identical rankings, and ‘2’,’3’ … ‘7’ indicate re-ordering 2, 3 … 7 species that were next to each other. Additionally, ‘2*’ indicates a special case where two pairs of species are inverted at different ends of the ranking scale. Taxon abbreviations: Ot, *Osteolaemus tetraspis*; Cm, *Crocodylus moreletii*; Cng, *Crocodylus novaeguineae*; Ci, *Crocodylus intermedius*; Cj, *Crocodylus johnstoni*; Mc, *Mecistops cataphractus*; Ts,*Tomistoma schlegelii*.

10.7717/peerj.204/supp-3 Figure S3 Percentage difference between volume- and surface-scaled models Absolute percentage differences between volume- and surface-scaled models. Columns are individually colour coded according to the highest and lowest differences for each species using the inbuilt conditional formatting function in Excel. Hot colours (red and orange) indicate large differences (the largest in red) while cooler colours (green and yellow) indicate smaller differences (the smallest in green). Note that the largest differences consistently occur under twisting load cases. Taxon abbreviations: Ot, *Osteolaemus tetraspis*; Cm, *Crocodylus moreletii*; Cng, *Crocodylus novaeguineae*; Ci, *Crocodylus intermedius*; Cj, *Crocodylus johnstoni*; Mc, *Mecistops cataphractus*; Ts,*Tomistoma schlegelii*.

10.7717/peerj.204/supp-4 Figure S4 Percentage difference between volume- and length-scaled models Absolute percentage differences between volume- and length-scaled models. Columns are individually colour coded according to the highest and lowest differences for each species using the inbuilt conditional formatting function in Excel. Hot colours (red and orange) indicate large differences (the largest in red) while cooler colours (green and yellow) indicate smaller differences (the smallest in green). Note that the largest differences consistently occur under twisting load cases. Taxon abbreviations: Ot, *Osteolaemus tetraspis*; Cm, *Crocodylus moreletii*; Cng, *Crocodylus novaeguineae*; Ci, *Crocodylus intermedius*; Cj, *Crocodylus johnstoni*; Mc, *Mecistops cataphractus*; Ts, *Tomistoma schlegelii*.

10.7717/peerj.204/supp-5 Figure S5 Percentage difference between surface- and length-scaled models Absolute percentage differences between surface- and length-scaled models. Columns are individually colour coded according to the highest and lowest differences for each species using the inbuilt conditional formatting function in Excel. Hot colours (red and orange) indicate large differences (the largest in red) while cooler colours (green and yellow) indicate smaller differences (the smallest in green). Note that the largest differences occur under twisting simulations for most species, with the exclusion of *C. intermedius*, which displays very small differences across all simulations. Taxon abbreviations: Ot, *Osteolaemus tetraspis*; Cm, *Crocodylus moreletii*; Cng, *Crocodylus novaeguineae*; Ci, *Crocodylus intermedius*; Cj, *Crocodylus johnstoni*; Mc, *Mecistops cataphractus*; Ts, *Tomistoma schlegelii*.

10.7717/peerj.204/supp-6 Figure S6 Standard Pattern Difference between volume- and surface-scaled simulations Response is standardised for each species with respect to *M. cataphractus* for volume- and surface-scaled simulation *conditions*, and the difference is then plotted for each *condition*. For an individual species a difference of zero indicates that it performs exactly the same (relative to *M. cataphractus*) for volume- and surface-scaling under that *condition*; and conversely, large deviations from zero indicate large differences in relative performance. (A) Orders *conditions* (left to right) by consistency in rank predictions, and (B) orders *conditions* (left to right) from the smallest average SPD through to the largest. For each *condition*, comparisons between ranked order is indicated by numbers, where ‘1’ (also marked by red stars) indicates identical rankings, and ‘2’, ’3’ … ‘7’ indicate re-ordering 2, 3 … 7 species that were next to each other. Additionally, ‘2*’ indicates a special case where two pairs of species are inverted at different ends of the ranking scale. Taxon abbreviations: Ot, *Osteolaemus tetraspis*; Cm, *Crocodylus moreletii*; Cng, *Crocodylus novaeguineae*; Ci, *Crocodylus intermedius*; Cj, *Crocodylus johnstoni*; Mc, *Mecistops cataphractus*; Ts, *Tomistoma schlegelii*.

10.7717/peerj.204/supp-7 Figure S7 Standard Pattern Difference between volume- and length-scaled simulations Response is standardised for each species with respect to *M. cataphractus* for volume- and length-scaled simulation *conditions*, and the difference is then plotted for each *condition*. For an individual species a difference of zero indicates that it performs exactly the same (relative to *M. cataphractus*) for volume- and length-scaling under that *condition*; and conversely, large deviations from zero indicate large differences in relative performance. (A) Orders *conditions* (left to right) by consistency in rank predictions, and (B) orders *conditions* (left to right) from the smallest average SPD through to the largest. For each *condition*, comparisons between ranked order is indicated by numbers, where ‘1’ (also marked by red stars) indicates identical rankings, and ‘2’, ’3’ … ‘7’ indicate re-ordering 2, 3 … 7 species that were next to each other. Additionally, ‘2*’ indicates a special case where two pairs of species are inverted at different ends of the ranking scale. Taxon abbreviations: Ot, *Osteolaemus tetraspis*; Cm, *Crocodylus moreletii*; Cng, *Crocodylus novaeguineae*; Ci, *Crocodylus intermedius*; Cj, *Crocodylus johnstoni*; Mc, *Mecistops cataphractus*; Ts, *Tomistoma schlegelii*.

10.7717/peerj.204/supp-8 Figure S8 Standard Pattern Difference between surface- and length-scaled simulations Response is standardised for each species with respect to *M. cataphractus* for surface- and length-scaled simulation *conditions*, and the difference is then plotted for each *condition*. For an individual species a difference of zero indicates that it performs exactly the same (relative to *M. cataphractus*) for surface- and length-scaling under that *condition*; and conversely, large deviations from zero indicate large differences in relative performance. (A) Orders *conditions* (left to right) by consistency in rank predictions, and (B) orders *conditions* (left to right) from the smallest average SPD through to the largest. For each *condition*, comparisons between ranked order is indicated by numbers, where ‘1’ (also marked by red stars) indicates identical rankings, and ‘2’, ’3’ … ‘7’ indicate re-ordering 2, 3 … 7 species that were next to each other. Additionally, ‘2*’ indicates a special case where two pairs of species are inverted at different ends of the ranking scale. Taxon abbreviations: Ot, *Osteolaemus tetraspis*; Cm, *Crocodylus moreletii*; Cng, *Crocodylus novaeguineae*; Ci, *Crocodylus intermedius*; Cj, *Crocodylus johnstoni*; Mc, *Mecistops cataphractus*; Ts, *Tomistoma schlegelii*.

10.7717/peerj.204/supp-9 Figure S9 Percentage difference between Linear Load Cases Absolute percentage differences between Linear Load Cases. Columns are individually colour coded according to the highest and lowest differences for each species using the inbuilt conditional formatting function in Excel. Hot colours (red and orange) indicate large differences (the largest in red) while cooler colours (green and yellow) indicate smaller differences (the smallest in green). Note that the largest differences occur under length-scaled biting simulations for all species, with the exclusion of *M. cataphractus*, which displays no difference for all simulation *conditions*. Taxon abbreviations: Ot, *Osteolaemus tetraspis*; Cm, *Crocodylus moreletii*; Cng, *Crocodylus novaeguineae*; Ci, *Crocodylus intermedius*; Cj, *Crocodylus johnstoni*; Mc, *Mecistops cataphractus*; Ts, *Tomistoma schlegelii*.

10.7717/peerj.204/supp-10 Figure S10 Standard Pattern Difference between Linear Load Case simulations Response is standardised for each species with respect to *M. cataphractus* for TeT/MeM and NoLLC/ELA Linear Load Case simulation *conditions*, and the difference is then plotted for each *condition*. For an individual species a difference of zero indicates that it performs exactly the same (relative to *M. cataphractus*) for TeT/MeM and NoLLC/ELA Linear Load Cases under that *condition*; and conversely, large deviations from zero indicate large differences in relative performance. (A) Orders *conditions* (left to right) by consistency in rank predictions, and (B) orders *conditions* (left to right) from the smallest average SPD through to the largest. For each *condition*, comparisons between ranked order is indicated by numbers, where ‘1’ (also marked by red stars) indicates identical rankings, and ‘2’, ’3’ … ‘7’ indicate re-ordering 2, 3 … 7 species that were next to each other. Additionally, ‘2*’ indicates a special case where two pairs of species are inverted at different ends of the ranking scale. Taxon abbreviations: Ot, *Osteolaemus tetraspis*; Cm, *Crocodylus moreletii*; Cng, *Crocodylus novaeguineae*; Ci, *Crocodylus intermedius*; Cj, *Crocodylus johnstoni*; Mc, *Mecistops cataphractus*; Ts, *Tomistoma schlegelii*.

10.7717/peerj.204/supp-11 Figure S11 Percentage difference between front and mid tooth positions Absolute percentage difference between simulations at front and mid tooth positions. Columns are individually colour coded according to the highest and lowest differences for each species using the inbuilt conditional formatting function in Excel. Hot colours (red and orange) indicate large differences (the largest in red) while cooler colours (green and yellow) indicate smaller differences (the smallest in green). Note that for all species the smallest differences tend to occur under biting simulations. Taxon abbreviations: Ot, *Osteolaemus tetraspis*; Cm, *Crocodylus moreletii*; Cng, *Crocodylus novaeguineae*; Ci, *Crocodylus intermedius*; Cj, *Crocodylus johnstoni*; Mc, *Mecistops cataphractus*; Ts, *Tomistoma schlegelii*.

10.7717/peerj.204/supp-12 Figure S12 Percentage difference between front and back tooth positions Absolute percentage difference between simulations at front and back tooth positions. Columns are individually colour coded according to the highest and lowest differences for each species using the inbuilt conditional formatting function in Excel. Hot colours (red and orange) indicate large differences (the largest in red) while cooler colours (green and yellow) indicate smaller differences (the smallest in green). Note that for all species the smallest differences tend to occur under biting simulations, and the largest under shaking. Taxon abbreviations: *Osteolaemus tetraspis*; Cm, *Crocodylus moreletii*; Cng, *Crocodylus novaeguineae*; Ci, *Crocodylus intermedius*; Cj, *Crocodylus johnstoni*; Mc, *Mecistops cataphractus*; Ts, *Tomistoma schlegelii*.

10.7717/peerj.204/supp-13 Figure S13 Percentage difference between mid and back tooth positions Absolute percentage difference between simulations at mid and back tooth positions. Columns are individually colour coded according to the highest and lowest differences for each species using the inbuilt conditional formatting function in Excel. Hot colours (red and orange) indicate large differences (the largest in red) while cooler colours (green and yellow) indicate smaller differences (the smallest in green). Note that for all species the smallest differences tend to occur under biting simulations, and the largest under shaking. Taxon abbreviations: Ot, *Osteolaemus tetraspis*; Cm, *Crocodylus moreletii*; Cng, *Crocodylus novaeguineae*; Ci, *Crocodylus intermedius*; Cj, *Crocodylus johnstoni*; Mc, *Mecistops cataphractus*; Ts, *Tomistoma schlegelii*.

10.7717/peerj.204/supp-14 Figure S14 Standard Pattern Difference between front and mid tooth position simulations. Response is standardised for each species with respect to *M. cataphractus* for front and mid tooth simulation *conditions*, and the difference is then plotted for each *condition*. For an individual species a difference of zero indicates that it performs exactly the same (relative to *M. cataphractus*) for front and mid tooth positions under that *condition*; and conversely, large deviations from zero indicate large differences in relative performance. (A) Orders *conditions* (left to right) by consistency in rank predictions, and (B) orders *conditions* (left to right) from the smallest average SPD through to the largest. For each *condition*, comparisons between ranked order is indicated by numbers, where ‘1’ (also marked by red stars) indicates identical rankings, and ‘2’, ’3’ … ‘7’ indicate re-ordering 2, 3 … 7 species that were next to each other. Additionally, ‘2*’ indicates a special case where two pairs of species are inverted at different ends of the ranking scale. Taxon abbreviations: Ot, *Osteolaemus tetraspis*; Cm, *Crocodylus moreletii*; Cng, *Crocodylus novaeguineae*; Ci, *Crocodylus intermedius*; Cj, *Crocodylus johnstoni*; Mc, *Mecistops cataphractus*; Ts, *Tomistoma schlegelii*.

10.7717/peerj.204/supp-15 Figure S15 Standard Pattern Difference between front and back tooth position simulations Response is standardised for each species with respect to *M. cataphractus* for front and back tooth simulation *conditions*, and the difference is then plotted for each *condition*. For an individual species a difference of zero indicates that it performs exactly the same (relative to *M. cataphractus*) for front and back tooth positions under that *condition*; and conversely, large deviations from zero indicate large differences in relative performance. (A) Orders *conditions* (left to right) by consistency in rank predictions, and (B) orders *conditions* (left to right) from the smallest average SPD through to the largest. For each *condition*, comparisons between ranked order is indicated by numbers, where ‘1’ (also marked by red stars) indicates identical rankings, and ‘2’,’3’ … ‘7’ indicate re-ordering 2, 3 … 7 species that were next to each other. Additionally, ‘2*’ indicates a special case where two pairs of species are inverted at different ends of the ranking scale. Taxon abbreviations: Ot, *Osteolaemus tetraspis*; Cm, *Crocodylus moreletii*; Cng, *Crocodylus novaeguineae*; Ci, *Crocodylus intermedius*; Cj, *Crocodylus johnstoni*; Mc, *Mecistops cataphractus*; Ts, *Tomistoma schlegelii*.

10.7717/peerj.204/supp-16 Figure S16 Standard Pattern Difference between mid and back tooth position simulations Response is standardised for each species with respect to *M. cataphractus* for mid and back tooth simulation *conditions*, and the difference is then plotted for each *condition*. For an individual species a difference of zero indicates that it performs exactly the same (relative to *M. cataphractus*) for mid and back tooth positions under that *condition*; and conversely, large deviations from zero indicate large differences in relative performance. (A) Orders *conditions* (left to right) by consistency in rank predictions, and (B) orders *conditions* (left to right) from the smallest average SPD through to the largest. For each *condition*, comparisons between ranked order is indicated by numbers, where ‘1’ (also marked by red stars) indicates identical rankings, and ‘2’, ’3’ … ‘7’ indicate re-ordering 2, 3 … 7 species that were next to each other. Additionally, ‘2*’ indicates a special case where two pairs of species are inverted at different ends of the ranking scale. Taxon abbreviations: Ot, *Osteolaemus tetraspis*; Cm, *Crocodylus moreletii*; Cng, *Crocodylus novaeguineae*; Ci, *Crocodylus intermedius*; Cj, *Crocodylus johnstoni*; Mc, *Mecistops cataphractus*; Ts, *Tomistoma schlegelii*.

10.7717/peerj.204/supp-17 Figure S17 Min, Max, and Mean percentage differences for individual species The range of percentage differences for each species is indicated by the upper (maximum % difference) and lower (minimum % difference) extent of vertical bars. These are shown for each of the modelling factor comparisons - which are ordered left to right based on the aggregated average of each comparison. Overall (A) includes differences from all feeding behaviours, while bite (B), shake (C), and twist (D) only include differences from their respective feeding types. Note that the order of modelling factor comparisons changes between biting (B), shaking (C), and twisting (D), suggesting that different feeding types are more (or less) sensitive to different modelling factors. Taxon abbreviations: Ot, *Osteolaemus tetraspis*; Cm, *Crocodylus moreletii*; Cng, *Crocodylus novaeguineae*; Ci, *Crocodylus intermedius*; Cj, *Crocodylus johnstoni*; Mc, *Mecistops cataphractus*; Ts, *Tomistoma schlegelii*.

10.7717/peerj.204/supp-18 Table S1 PC and ΔPC Values Reported values for the first two principal components (labelled PC1 and PC2) are from Walmsley et al. (2013). Between them they account for 92% of shape variation within this study group (66% PC1, 26% PC2). ΔPC1 and ΔPC2 are calculated as the difference in shape between each species model (Taxon) to that of *Mecistops cataphractus* for PC1 and PC2 respectively; these ΔPC scores are effectively a measure of relative difference in the shape of each species to that of *Mecistops cataphractus*.
