# Supplementary figures and images for: Beware the black box: investigating the sensitivity of FEA simulations to modelling factors in comparative biomechanics
Source: PeerJ. 2013 Nov 5;1:e204. doi: 10.7717/peerj.204 (PMC3828634; doi:10.7717/peerj.204)

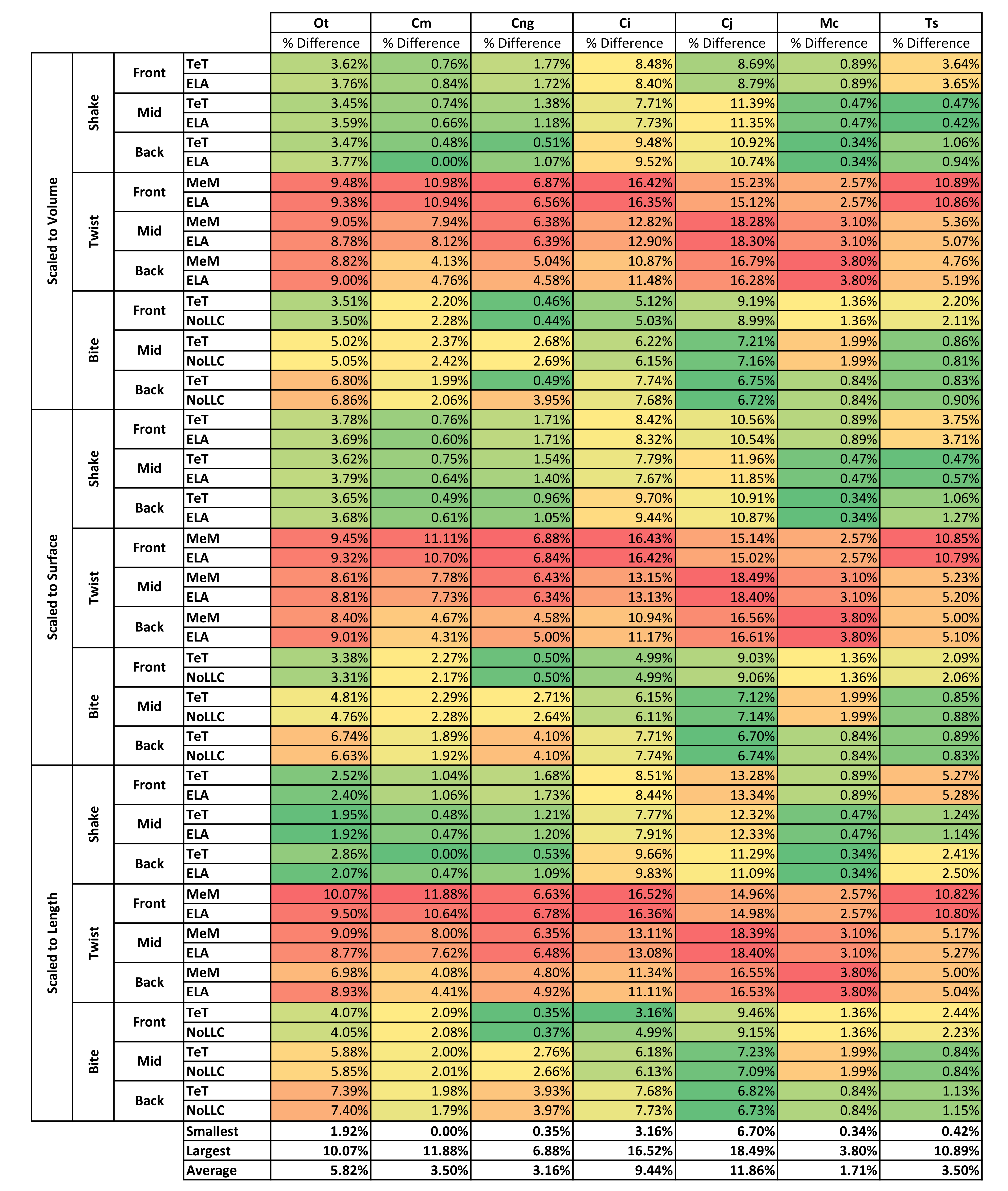

Supplement: Figure S1 — Absolute percentage difference between models simulated with HET and HOM material properties. Columns are individually colour coded according to the highest and lowest differences for that species using the inbuilt conditional formatting function in Excel. Hot colours (red and orange) indicate large differences (the largest in red) while cooler colours (green and yellow) indicate smaller differences (the smallest in green). Note that the largest differences consistently occur under twisting load cases. Taxon abbreviations: Ot, Osteolaemus tetraspis; Cm, Crocodylus moreletii; Cng, Crocodylus novaeguineae; Ci, Crocodylus intermedius; Cj, Crocodylus johnstoni; Mc, Mecistops cataphractus; Ts,Tomistoma schlegelii. [file peerj-01-204-s001.png]

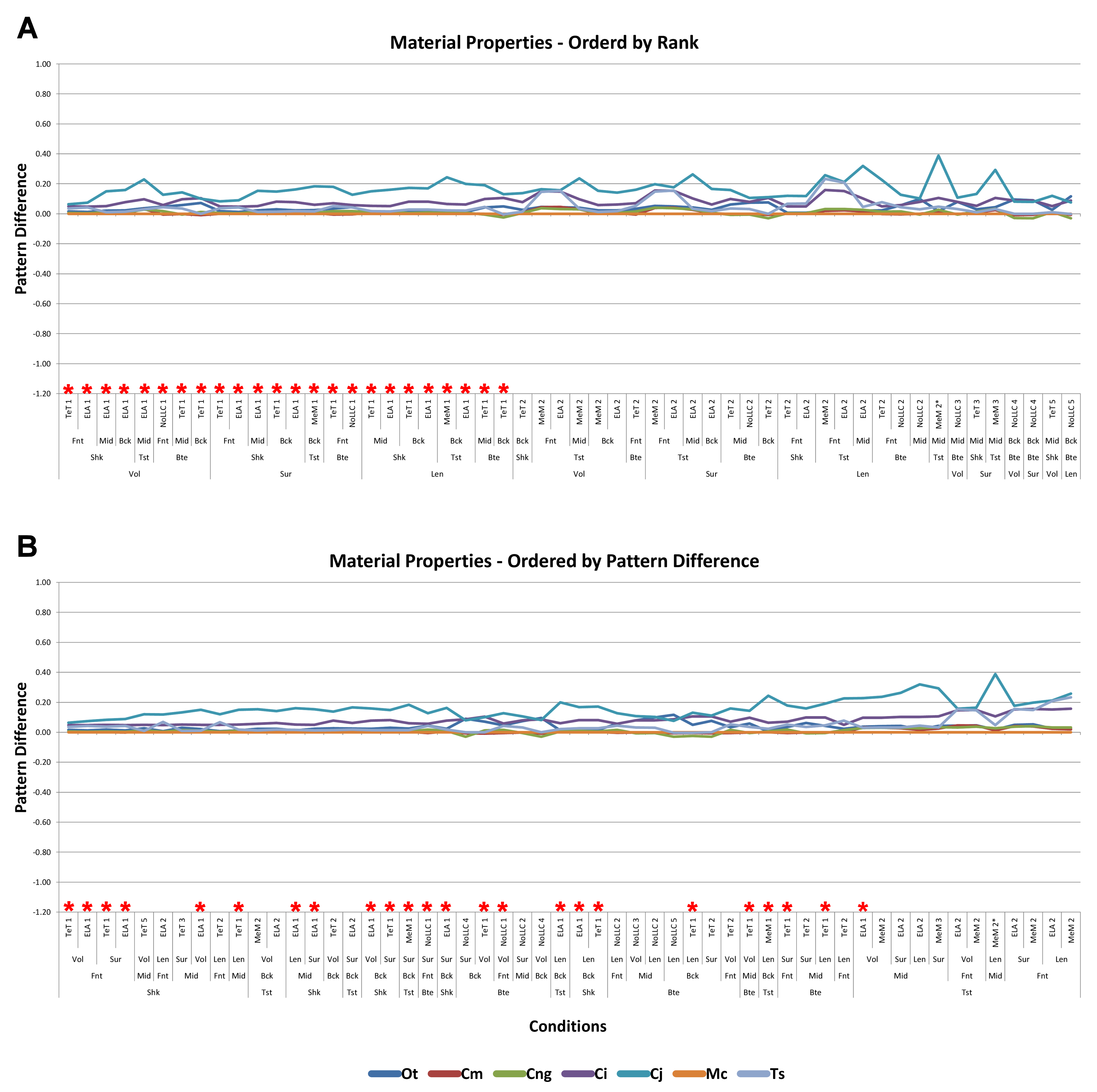

Supplement: Figure S2 — Response is standardised for each species with respect to M. cataphractus for HET and HOM simulation conditions, and the difference is then plotted for each condition. For an individual species a difference of zero indicates that it performs exactly the same (relative to M. cataphractus) for HET and HOM material properties under that condition; and conversely, large deviations from zero indicate large differences in relative performance. (A) Orders conditions (left to right) by consistency in rank predictions, and (B) orders conditions (left to right) from the smallest average SPD through to the largest. For each condition, comparisons between ranked order is indicated by numbers, where ‘1’ (also marked by red stars) indicates identical rankings, and ‘2’,’3’ … ‘7’ indicate re-ordering 2, 3 … 7 species that were next to each other. Additionally, ‘2*’ indicates a special case where two pairs of species are inverted at different ends of the ranking scale. Taxon abbreviations: Ot, Osteolaemus tetraspis; Cm, Crocodylus moreletii; Cng, Crocodylus novaeguineae; Ci, Crocodylus intermedius; Cj, Crocodylus johnstoni; Mc, Mecistops cataphractus; Ts,Tomistoma schlegelii. [file peerj-01-204-s002.png]

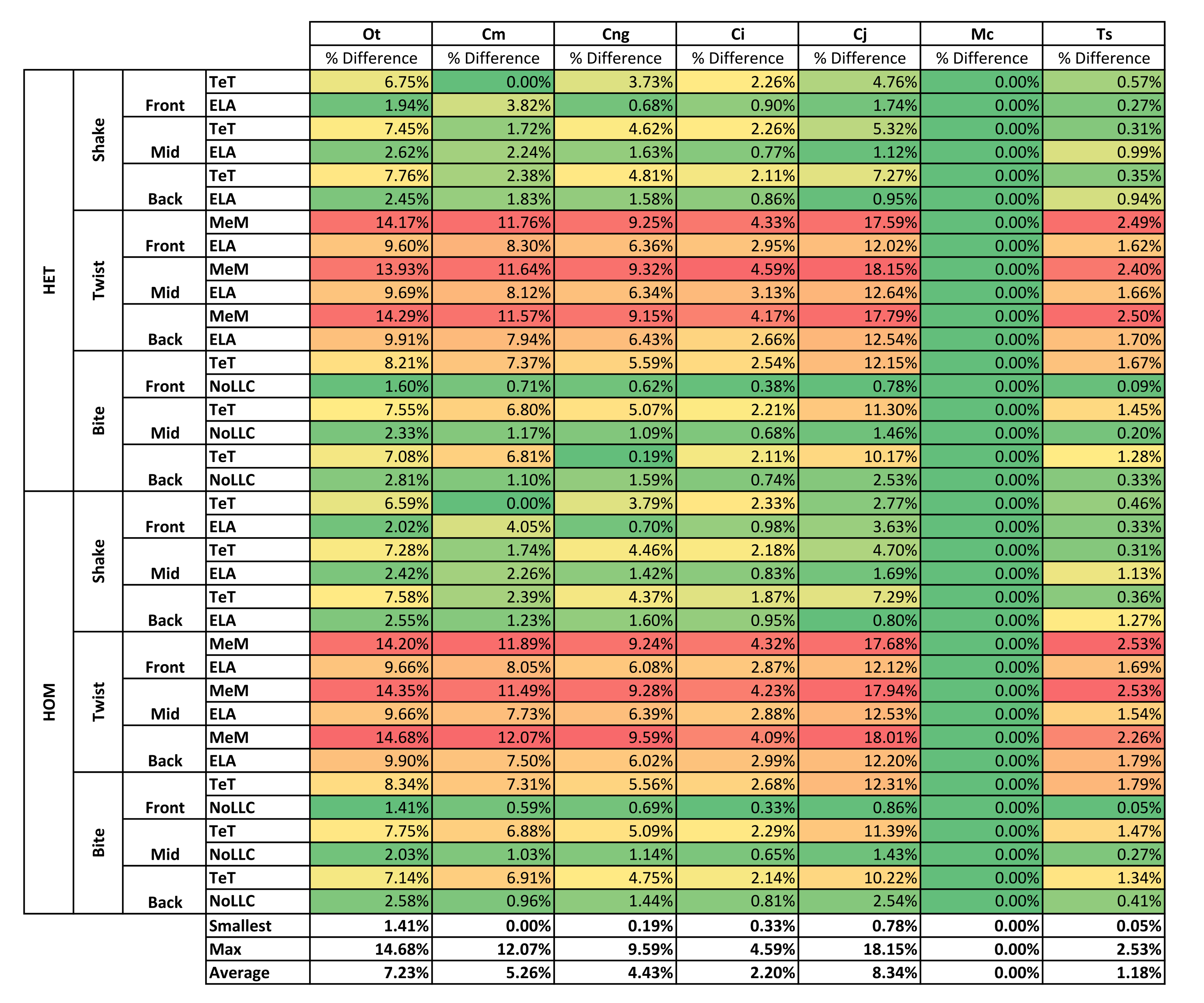

Supplement: Figure S3 — Absolute percentage differences between volume- and surface-scaled models. Columns are individually colour coded according to the highest and lowest differences for each species using the inbuilt conditional formatting function in Excel. Hot colours (red and orange) indicate large differences (the largest in red) while cooler colours (green and yellow) indicate smaller differences (the smallest in green). Note that the largest differences consistently occur under twisting load cases. Taxon abbreviations: Ot, Osteolaemus tetraspis; Cm, Crocodylus moreletii; Cng, Crocodylus novaeguineae; Ci, Crocodylus intermedius; Cj, Crocodylus johnstoni; Mc, Mecistops cataphractus; Ts,Tomistoma schlegelii. [file peerj-01-204-s003.png]

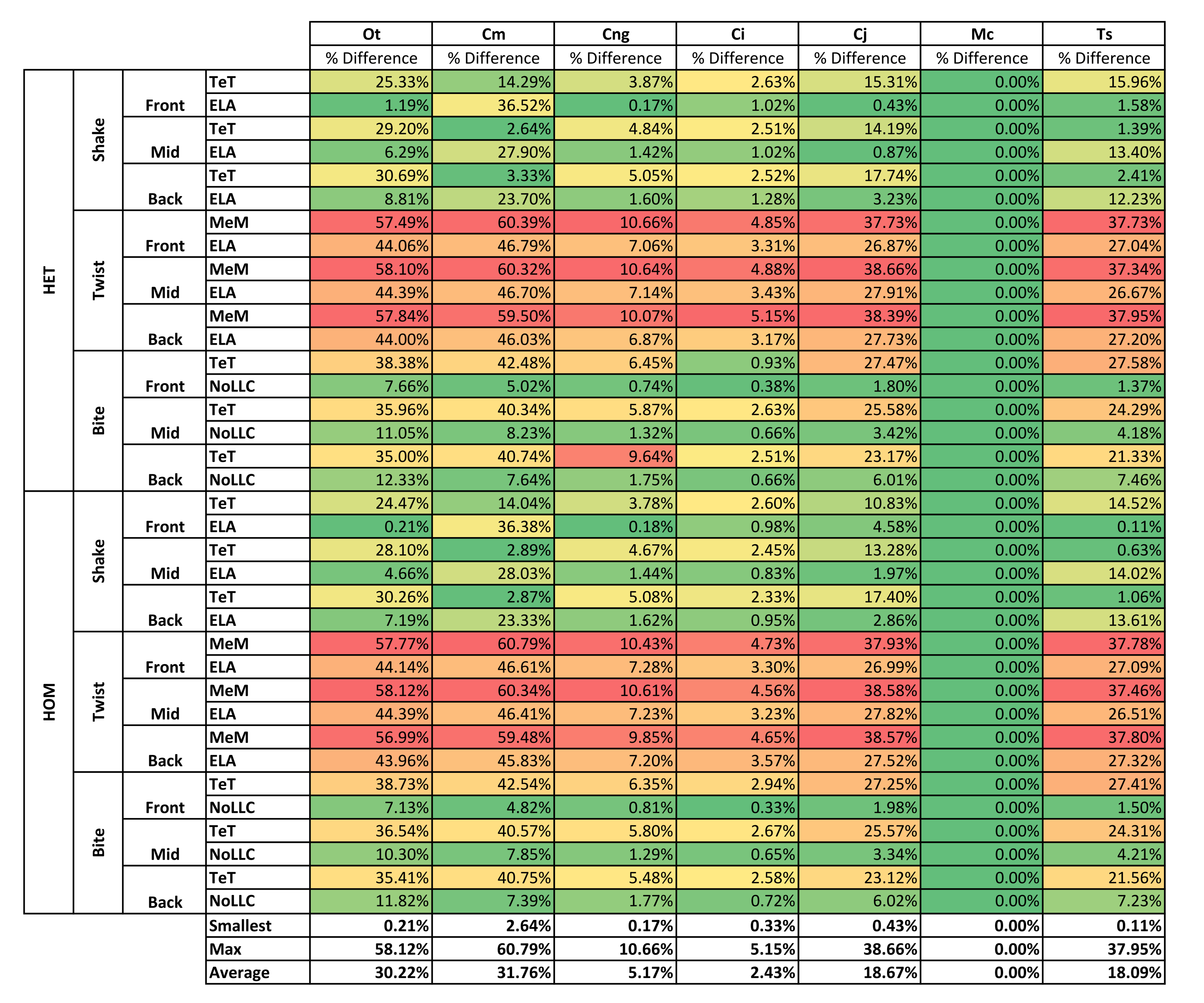

Supplement: Figure S4 — Absolute percentage differences between volume- and length-scaled models. Columns are individually colour coded according to the highest and lowest differences for each species using the inbuilt conditional formatting function in Excel. Hot colours (red and orange) indicate large differences (the largest in red) while cooler colours (green and yellow) indicate smaller differences (the smallest in green). Note that the largest differences consistently occur under twisting load cases. Taxon abbreviations: Ot, Osteolaemus tetraspis; Cm, Crocodylus moreletii; Cng, Crocodylus novaeguineae; Ci, Crocodylus intermedius; Cj, Crocodylus johnstoni; Mc, Mecistops cataphractus; Ts, Tomistoma schlegelii. [file peerj-01-204-s004.png]

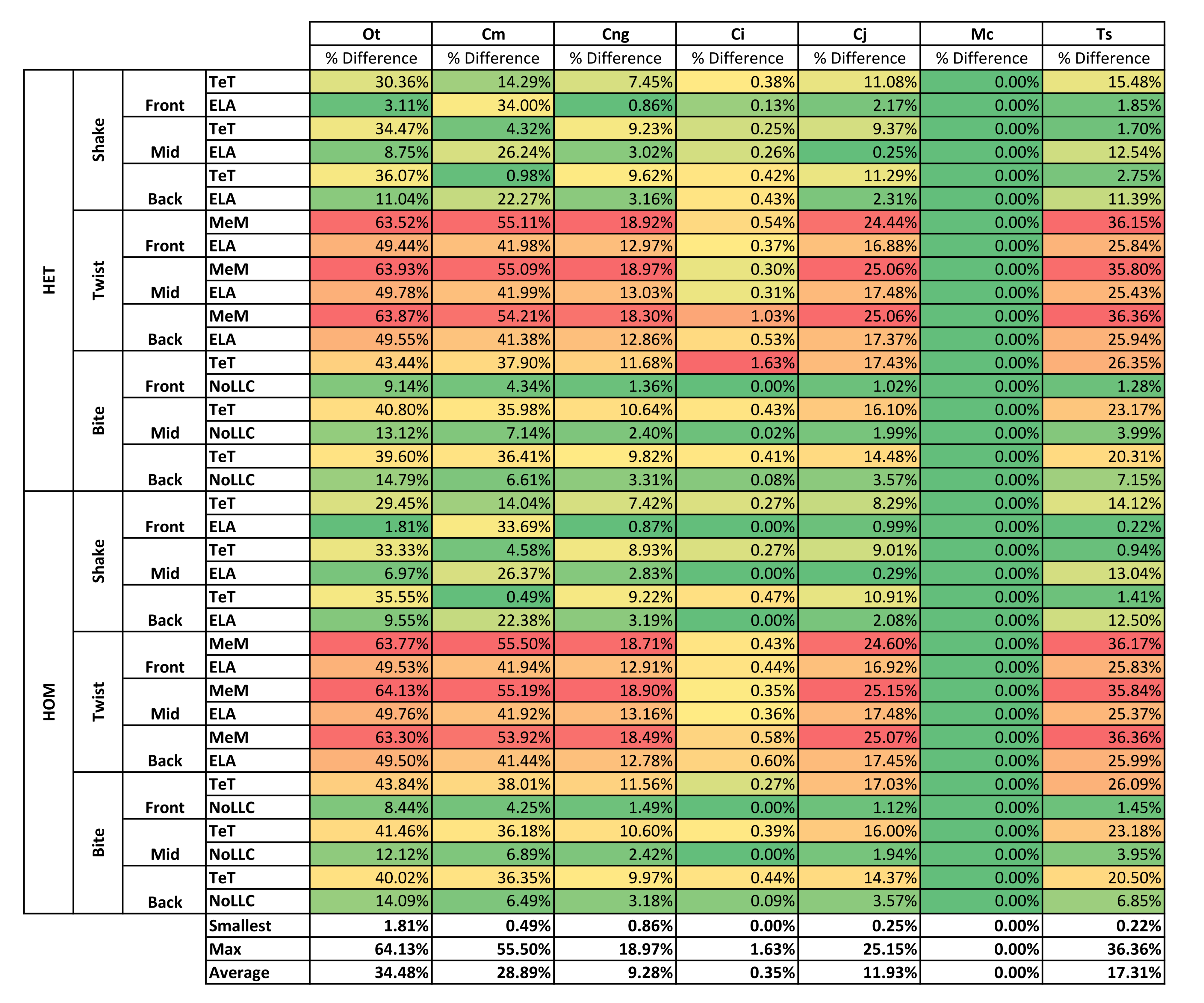

Supplement: Figure S5 — Absolute percentage differences between surface- and length-scaled models. Columns are individually colour coded according to the highest and lowest differences for each species using the inbuilt conditional formatting function in Excel. Hot colours (red and orange) indicate large differences (the largest in red) while cooler colours (green and yellow) indicate smaller differences (the smallest in green). Note that the largest differences occur under twisting simulations for most species, with the exclusion of C. intermedius, which displays very small differences across all simulations. Taxon abbreviations: Ot, Osteolaemus tetraspis; Cm, Crocodylus moreletii; Cng, Crocodylus novaeguineae; Ci, Crocodylus intermedius; Cj, Crocodylus johnstoni; Mc, Mecistops cataphractus; Ts, Tomistoma schlegelii. [file peerj-01-204-s005.png]

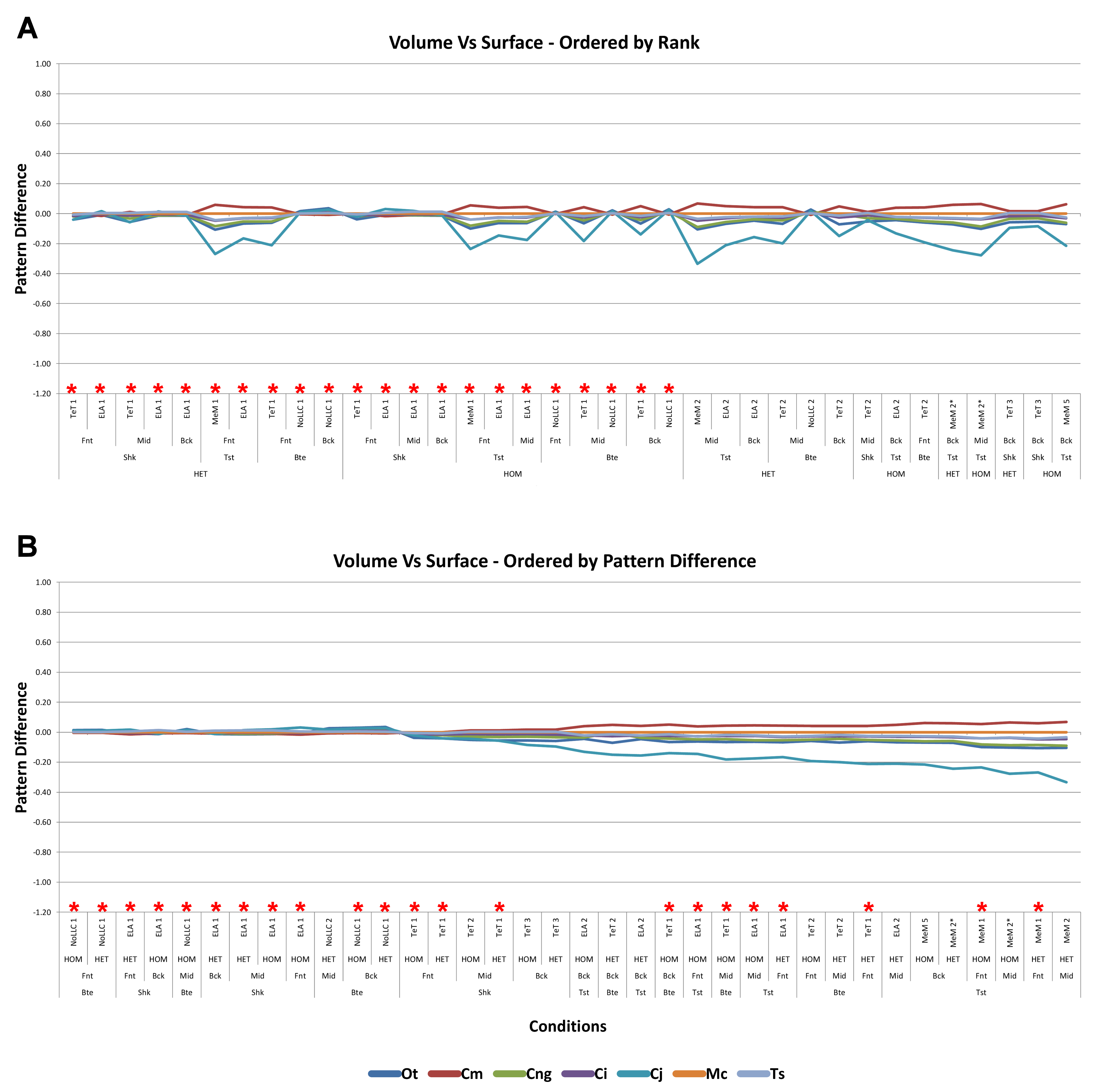

Supplement: Figure S6 — Response is standardised for each species with respect to M. cataphractus for volume- and surface-scaled simulation conditions, and the difference is then plotted for each condition. For an individual species a difference of zero indicates that it performs exactly the same (relative to M. cataphractus) for volume- and surface-scaling under that condition; and conversely, large deviations from zero indicate large differences in relative performance. (A) Orders conditions (left to right) by consistency in rank predictions, and (B) orders conditions (left to right) from the smallest average SPD through to the largest. For each condition, comparisons between ranked order is indicated by numbers, where ‘1’ (also marked by red stars) indicates identical rankings, and ‘2’, ’3’ … ‘7’ indicate re-ordering 2, 3 … 7 species that were next to each other. Additionally, ‘2*’ indicates a special case where two pairs of species are inverted at different ends of the ranking scale. Taxon abbreviations: Ot, Osteolaemus tetraspis; Cm, Crocodylus moreletii; Cng, Crocodylus novaeguineae; Ci, Crocodylus intermedius; Cj, Crocodylus johnstoni; Mc, Mecistops cataphractus; Ts, Tomistoma schlegelii. [file peerj-01-204-s006.png]

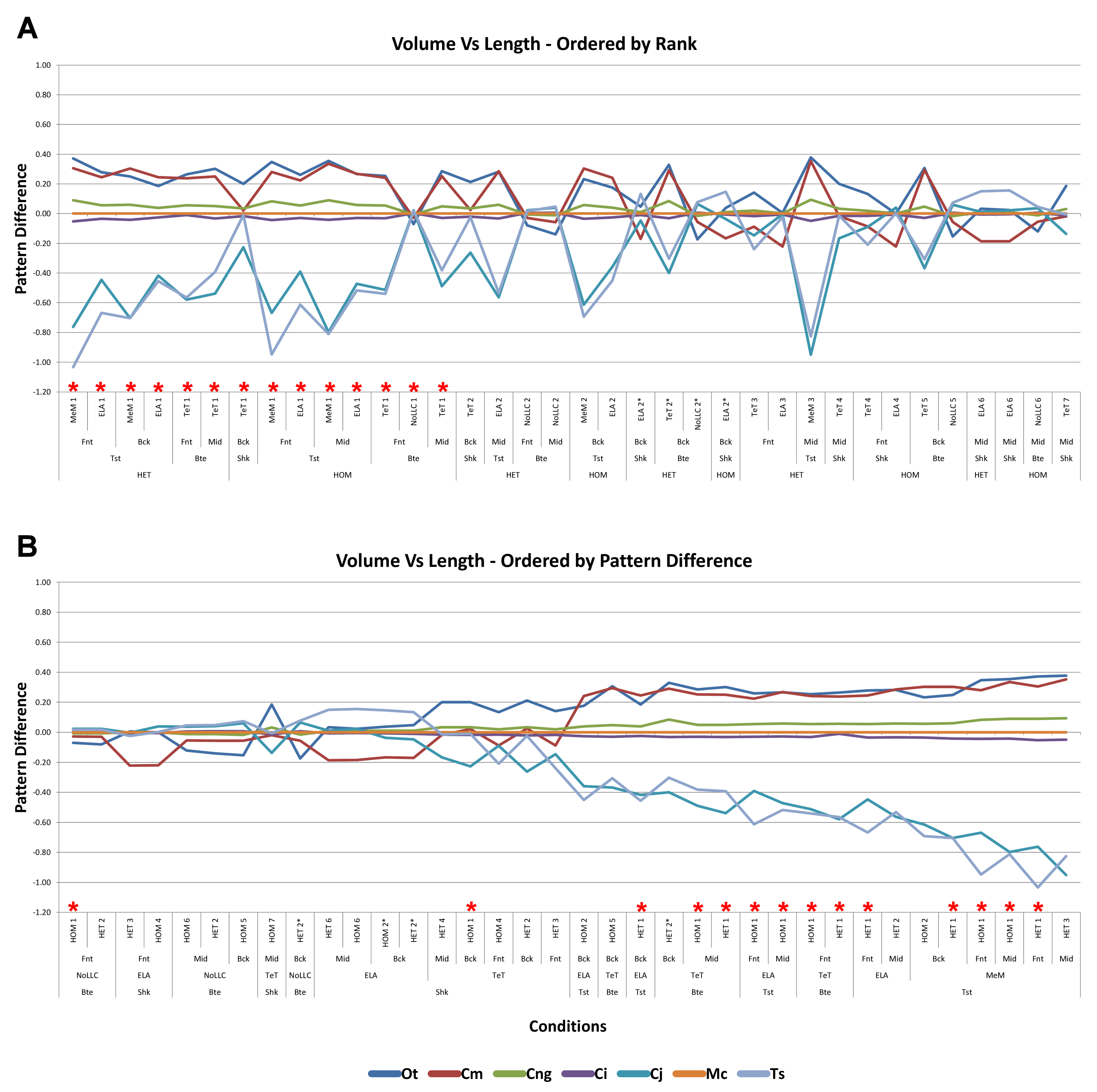

Supplement: Figure S7 — Response is standardised for each species with respect to M. cataphractus for volume- and length-scaled simulation conditions, and the difference is then plotted for each condition. For an individual species a difference of zero indicates that it performs exactly the same (relative to M. cataphractus) for volume- and length-scaling under that condition; and conversely, large deviations from zero indicate large differences in relative performance. (A) Orders conditions (left to right) by consistency in rank predictions, and (B) orders conditions (left to right) from the smallest average SPD through to the largest. For each condition, comparisons between ranked order is indicated by numbers, where ‘1’ (also marked by red stars) indicates identical rankings, and ‘2’, ’3’ … ‘7’ indicate re-ordering 2, 3 … 7 species that were next to each other. Additionally, ‘2*’ indicates a special case where two pairs of species are inverted at different ends of the ranking scale. Taxon abbreviations: Ot, Osteolaemus tetraspis; Cm, Crocodylus moreletii; Cng, Crocodylus novaeguineae; Ci, Crocodylus intermedius; Cj, Crocodylus johnstoni; Mc, Mecistops cataphractus; Ts, Tomistoma schlegelii. [file peerj-01-204-s007.png]

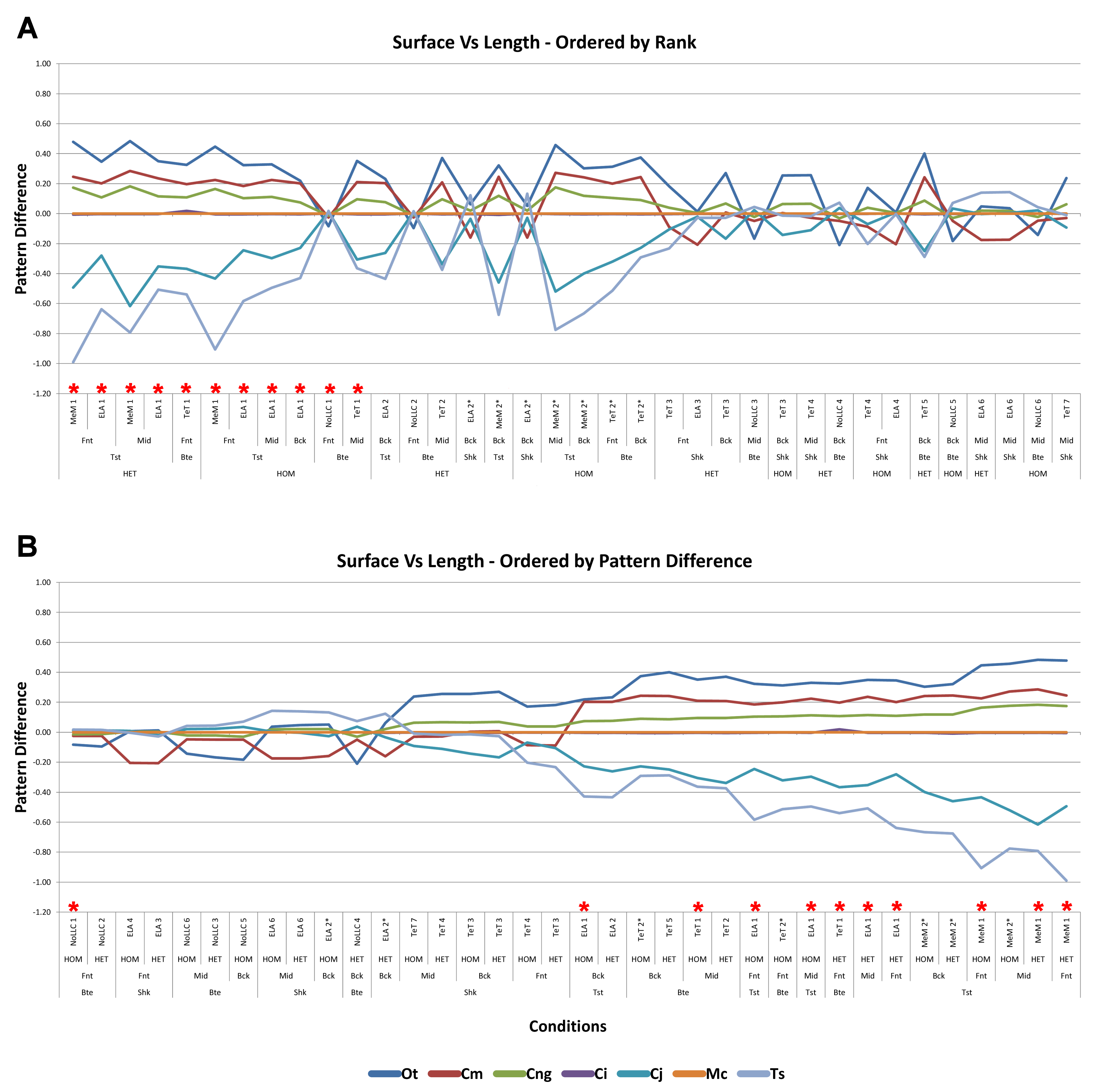

Supplement: Figure S8 — Response is standardised for each species with respect to M. cataphractus for surface- and length-scaled simulation conditions, and the difference is then plotted for each condition. For an individual species a difference of zero indicates that it performs exactly the same (relative to M. cataphractus) for surface- and length-scaling under that condition; and conversely, large deviations from zero indicate large differences in relative performance. (A) Orders conditions (left to right) by consistency in rank predictions, and (B) orders conditions (left to right) from the smallest average SPD through to the largest. For each condition, comparisons between ranked order is indicated by numbers, where ‘1’ (also marked by red stars) indicates identical rankings, and ‘2’, ’3’ … ‘7’ indicate re-ordering 2, 3 … 7 species that were next to each other. Additionally, ‘2*’ indicates a special case where two pairs of species are inverted at different ends of the ranking scale. Taxon abbreviations: Ot, Osteolaemus tetraspis; Cm, Crocodylus moreletii; Cng, Crocodylus novaeguineae; Ci, Crocodylus intermedius; Cj, Crocodylus johnstoni; Mc, Mecistops cataphractus; Ts, Tomistoma schlegelii. [file peerj-01-204-s008.png]

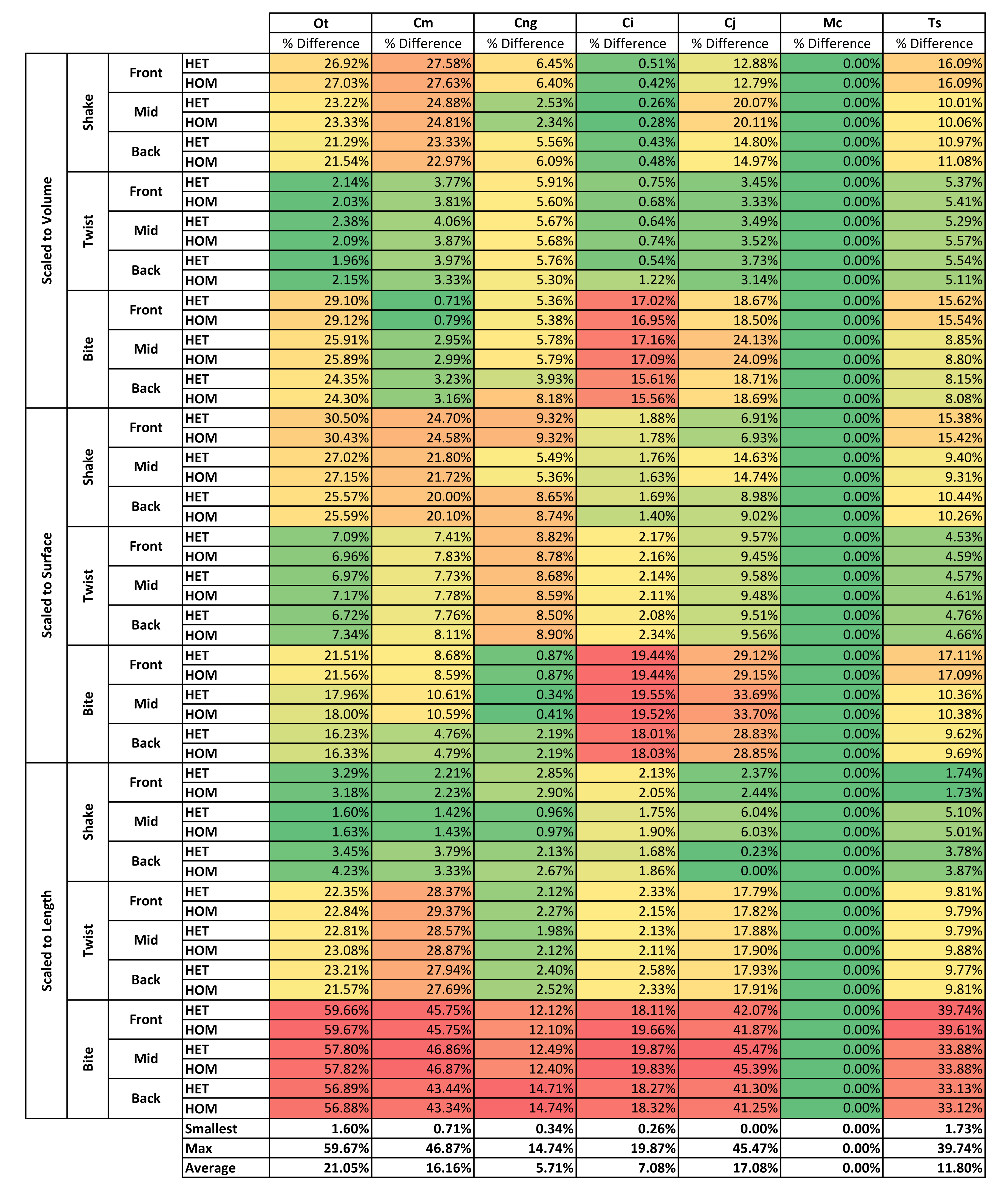

Supplement: Figure S9 — Absolute percentage differences between Linear Load Cases. Columns are individually colour coded according to the highest and lowest differences for each species using the inbuilt conditional formatting function in Excel. Hot colours (red and orange) indicate large differences (the largest in red) while cooler colours (green and yellow) indicate smaller differences (the smallest in green). Note that the largest differences occur under length-scaled biting simulations for all species, with the exclusion of M. cataphractus, which displays no difference for all simulation conditions. Taxon abbreviations: Ot, Osteolaemus tetraspis; Cm, Crocodylus moreletii; Cng, Crocodylus novaeguineae; Ci, Crocodylus intermedius; Cj, Crocodylus johnstoni; Mc, Mecistops cataphractus; Ts, Tomistoma schlegelii. [file peerj-01-204-s009.png]

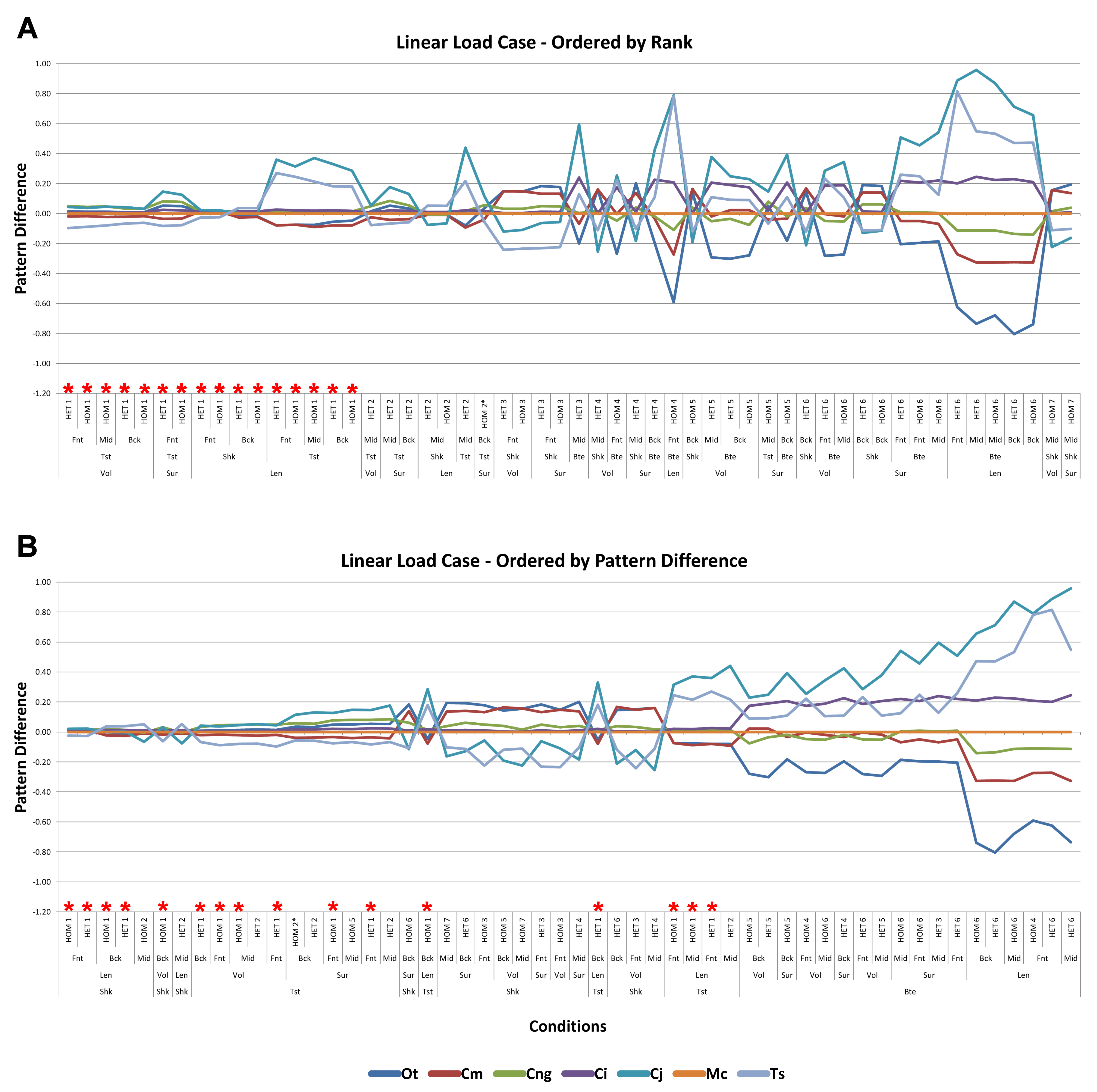

Supplement: Figure S10 — Response is standardised for each species with respect to M. cataphractus for TeT/MeM and NoLLC/ELA Linear Load Case simulation conditions, and the difference is then plotted for each condition. For an individual species a difference of zero indicates that it performs exactly the same (relative to M. cataphractus) for TeT/MeM and NoLLC/ELA Linear Load Cases under that condition; and conversely, large deviations from zero indicate large differences in relative performance. (A) Orders conditions (left to right) by consistency in rank predictions, and (B) orders conditions (left to right) from the smallest average SPD through to the largest. For each condition, comparisons between ranked order is indicated by numbers, where ‘1’ (also marked by red stars) indicates identical rankings, and ‘2’, ’3’ … ‘7’ indicate re-ordering 2, 3 … 7 species that were next to each other. Additionally, ‘2*’ indicates a special case where two pairs of species are inverted at different ends of the ranking scale. Taxon abbreviations: Ot, Osteolaemus tetraspis; Cm, Crocodylus moreletii; Cng, Crocodylus novaeguineae; Ci, Crocodylus intermedius; Cj, Crocodylus johnstoni; Mc, Mecistops cataphractus; Ts, Tomistoma schlegelii. [file peerj-01-204-s010.png]

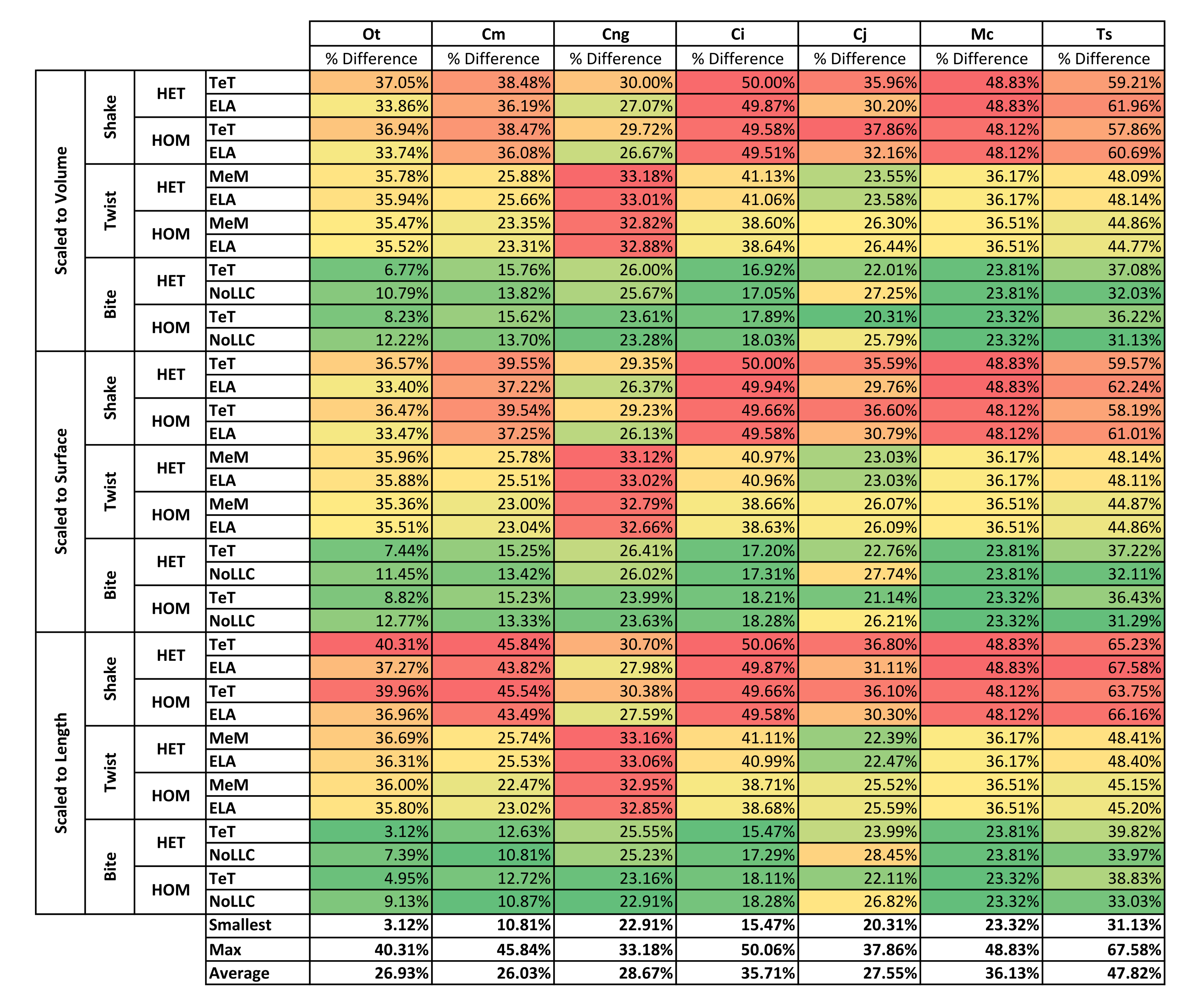

Supplement: Figure S11 — Absolute percentage difference between simulations at front and mid tooth positions. Columns are individually colour coded according to the highest and lowest differences for each species using the inbuilt conditional formatting function in Excel. Hot colours (red and orange) indicate large differences (the largest in red) while cooler colours (green and yellow) indicate smaller differences (the smallest in green). Note that for all species the smallest differences tend to occur under biting simulations. Taxon abbreviations: Ot, Osteolaemus tetraspis; Cm, Crocodylus moreletii; Cng, Crocodylus novaeguineae; Ci, Crocodylus intermedius; Cj, Crocodylus johnstoni; Mc, Mecistops cataphractus; Ts, Tomistoma schlegelii. [file peerj-01-204-s011.png]

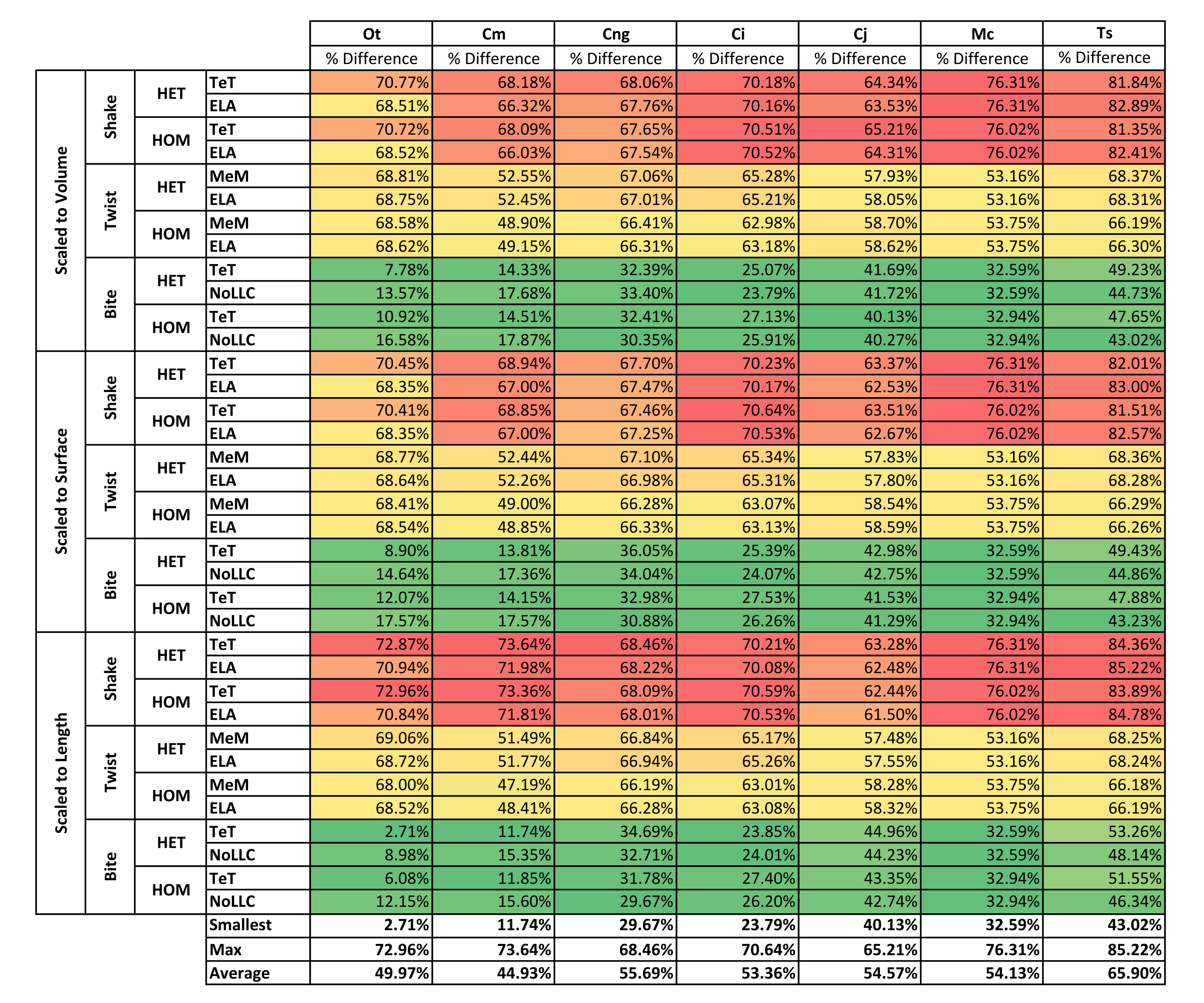

Supplement: Figure S12 — Absolute percentage difference between simulations at front and back tooth positions. Columns are individually colour coded according to the highest and lowest differences for each species using the inbuilt conditional formatting function in Excel. Hot colours (red and orange) indicate large differences (the largest in red) while cooler colours (green and yellow) indicate smaller differences (the smallest in green). Note that for all species the smallest differences tend to occur under biting simulations, and the largest under shaking. Taxon abbreviations: Osteolaemus tetraspis; Cm, Crocodylus moreletii; Cng, Crocodylus novaeguineae; Ci, Crocodylus intermedius; Cj, Crocodylus johnstoni; Mc, Mecistops cataphractus; Ts, Tomistoma schlegelii. [file peerj-01-204-s012.png]

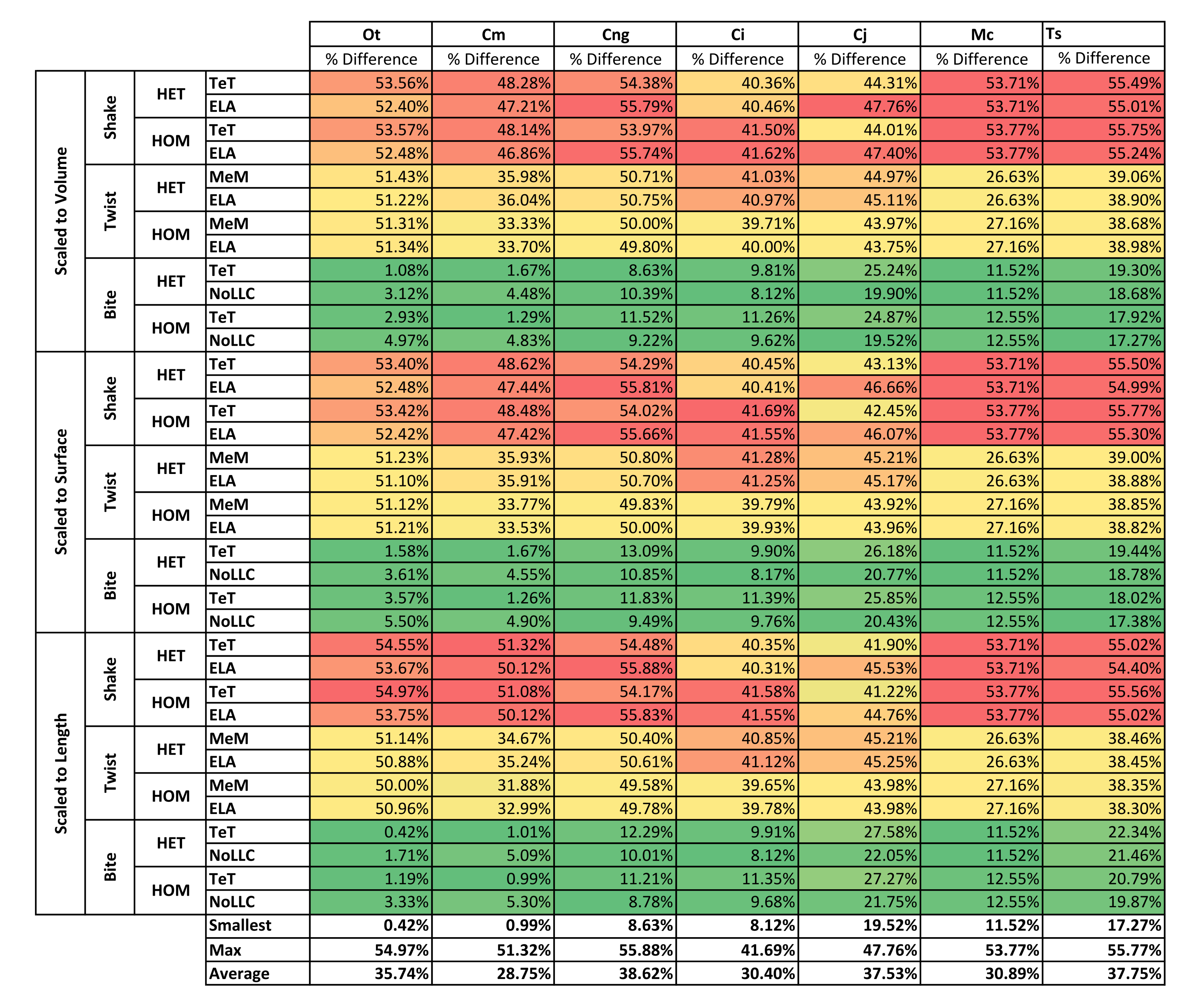

Supplement: Figure S13 — Absolute percentage difference between simulations at mid and back tooth positions. Columns are individually colour coded according to the highest and lowest differences for each species using the inbuilt conditional formatting function in Excel. Hot colours (red and orange) indicate large differences (the largest in red) while cooler colours (green and yellow) indicate smaller differences (the smallest in green). Note that for all species the smallest differences tend to occur under biting simulations, and the largest under shaking. Taxon abbreviations: Ot, Osteolaemus tetraspis; Cm, Crocodylus moreletii; Cng, Crocodylus novaeguineae; Ci, Crocodylus intermedius; Cj, Crocodylus johnstoni; Mc, Mecistops cataphractus; Ts, Tomistoma schlegelii. [file peerj-01-204-s013.png]

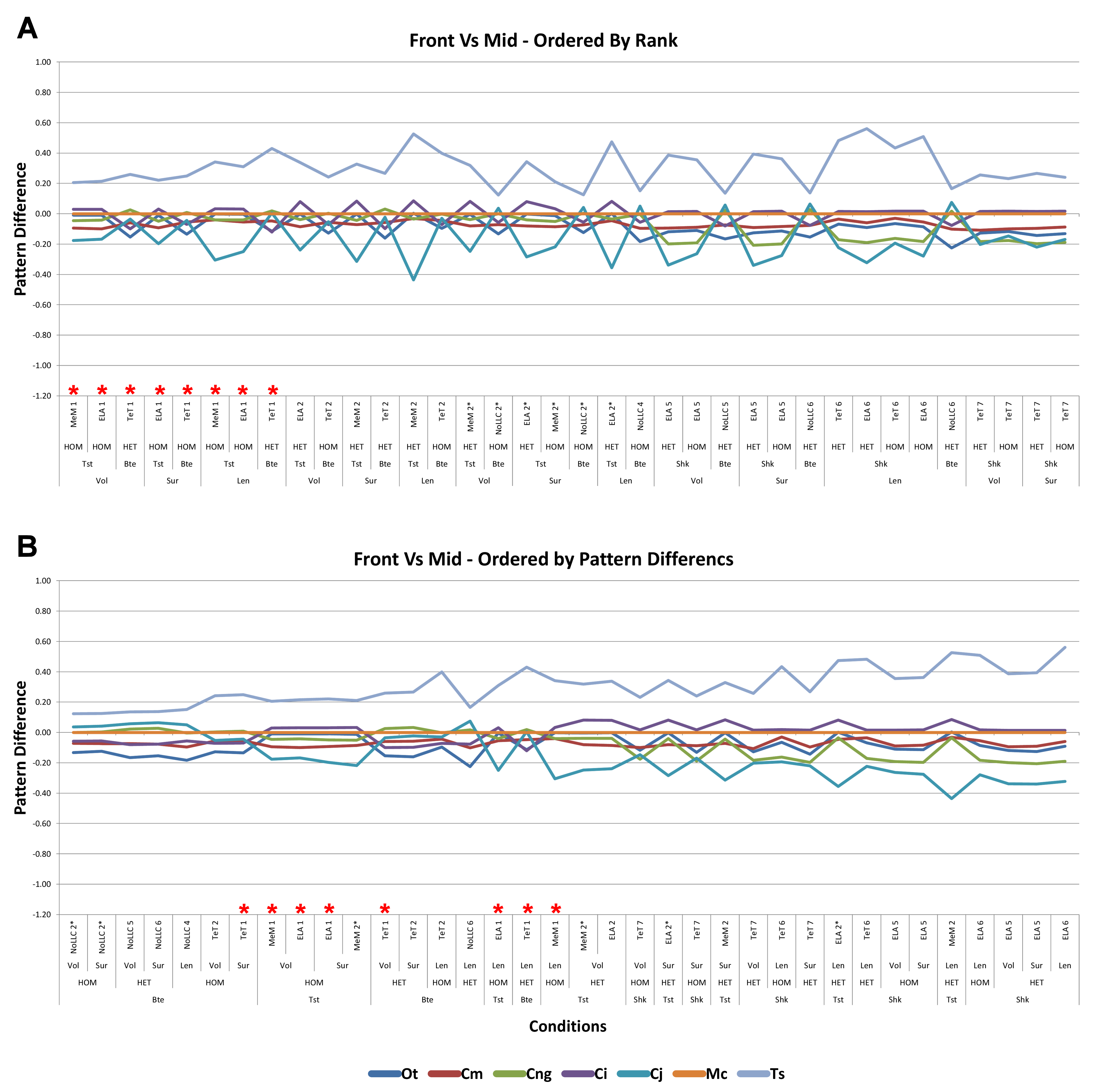

Supplement: Figure S14 — Response is standardised for each species with respect to M. cataphractus for front and mid tooth simulation conditions, and the difference is then plotted for each condition. For an individual species a difference of zero indicates that it performs exactly the same (relative to M. cataphractus) for front and mid tooth positions under that condition; and conversely, large deviations from zero indicate large differences in relative performance. (A) Orders conditions (left to right) by consistency in rank predictions, and (B) orders conditions (left to right) from the smallest average SPD through to the largest. For each condition, comparisons between ranked order is indicated by numbers, where ‘1’ (also marked by red stars) indicates identical rankings, and ‘2’, ’3’ … ‘7’ indicate re-ordering 2, 3 … 7 species that were next to each other. Additionally, ‘2*’ indicates a special case where two pairs of species are inverted at different ends of the ranking scale. Taxon abbreviations: Ot, Osteolaemus tetraspis; Cm, Crocodylus moreletii; Cng, Crocodylus novaeguineae; Ci, Crocodylus intermedius; Cj, Crocodylus johnstoni; Mc, Mecistops cataphractus; Ts, Tomistoma schlegelii. [file peerj-01-204-s014.png]

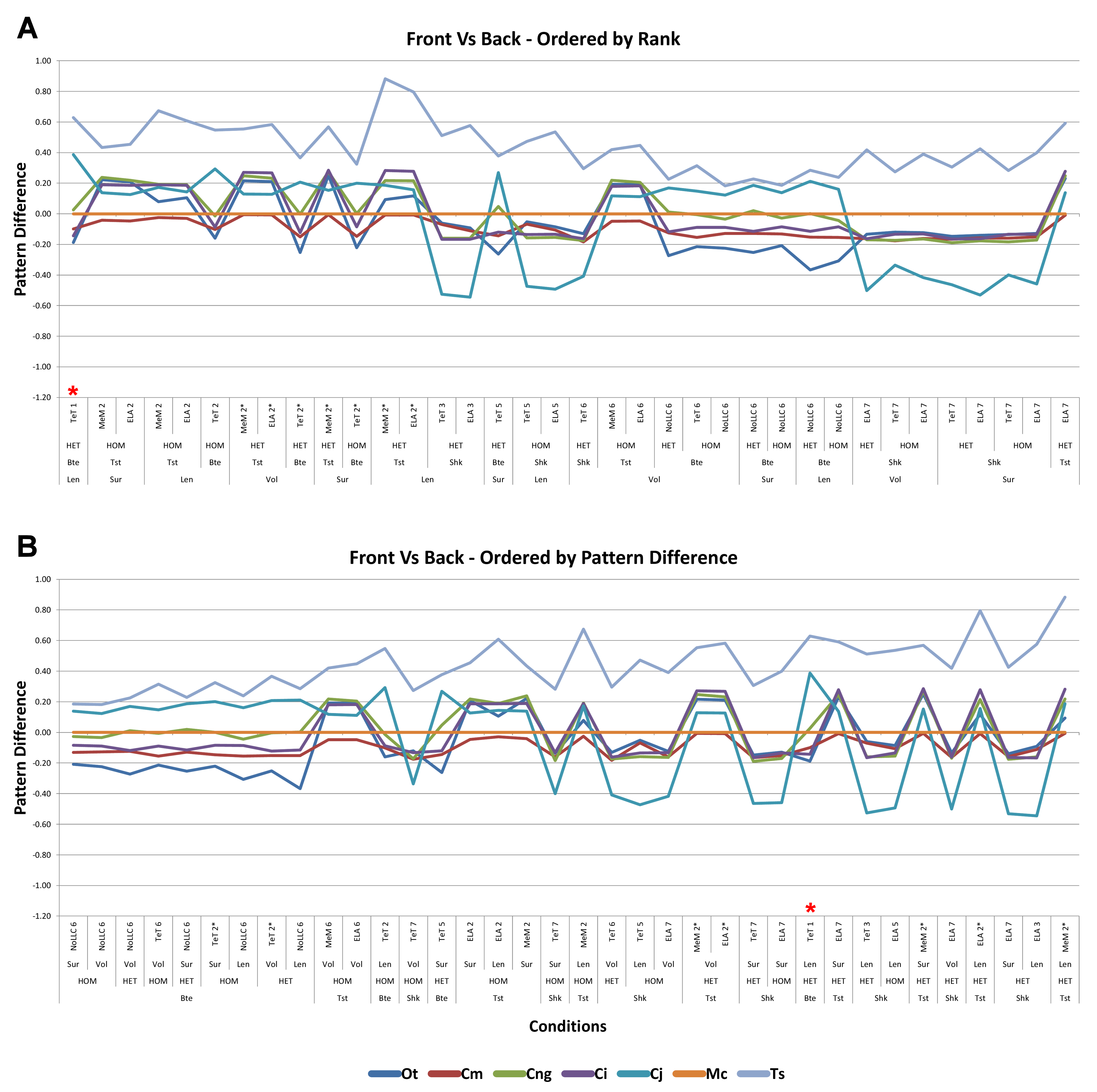

Supplement: Figure S15 — Response is standardised for each species with respect to M. cataphractus for front and back tooth simulation conditions, and the difference is then plotted for each condition. For an individual species a difference of zero indicates that it performs exactly the same (relative to M. cataphractus) for front and back tooth positions under that condition; and conversely, large deviations from zero indicate large differences in relative performance. (A) Orders conditions (left to right) by consistency in rank predictions, and (B) orders conditions (left to right) from the smallest average SPD through to the largest. For each condition, comparisons between ranked order is indicated by numbers, where ‘1’ (also marked by red stars) indicates identical rankings, and ‘2’,’3’ … ‘7’ indicate re-ordering 2, 3 … 7 species that were next to each other. Additionally, ‘2*’ indicates a special case where two pairs of species are inverted at different ends of the ranking scale. Taxon abbreviations: Ot, Osteolaemus tetraspis; Cm, Crocodylus moreletii; Cng, Crocodylus novaeguineae; Ci, Crocodylus intermedius; Cj, Crocodylus johnstoni; Mc, Mecistops cataphractus; Ts, Tomistoma schlegelii. [file peerj-01-204-s015.png]

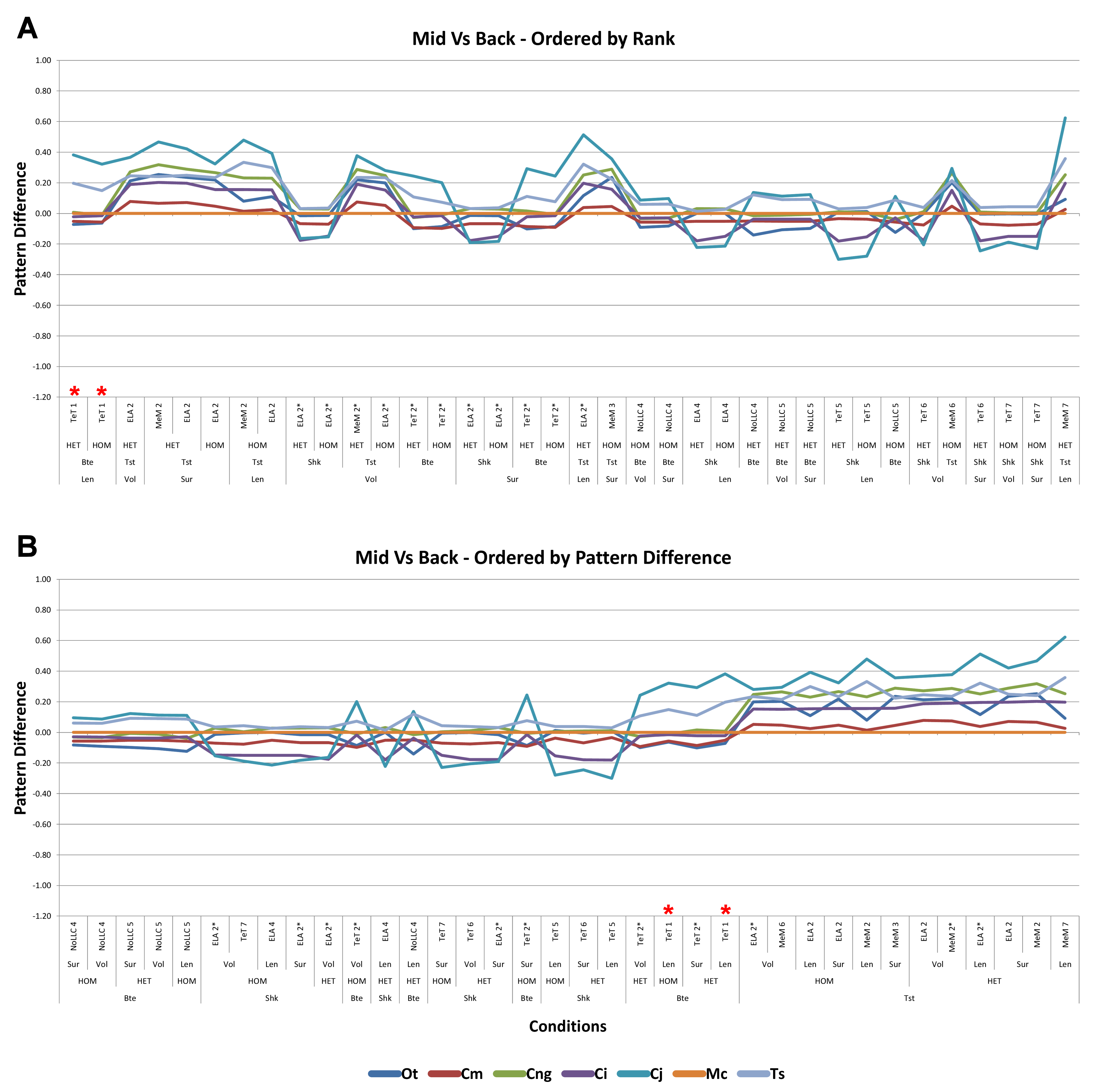

Supplement: Figure S16 — Response is standardised for each species with respect to M. cataphractus for mid and back tooth simulation conditions, and the difference is then plotted for each condition. For an individual species a difference of zero indicates that it performs exactly the same (relative to M. cataphractus) for mid and back tooth positions under that condition; and conversely, large deviations from zero indicate large differences in relative performance. (A) Orders conditions (left to right) by consistency in rank predictions, and (B) orders conditions (left to right) from the smallest average SPD through to the largest. For each condition, comparisons between ranked order is indicated by numbers, where ‘1’ (also marked by red stars) indicates identical rankings, and ‘2’, ’3’ … ‘7’ indicate re-ordering 2, 3 … 7 species that were next to each other. Additionally, ‘2*’ indicates a special case where two pairs of species are inverted at different ends of the ranking scale. Taxon abbreviations: Ot, Osteolaemus tetraspis; Cm, Crocodylus moreletii; Cng, Crocodylus novaeguineae; Ci, Crocodylus intermedius; Cj, Crocodylus johnstoni; Mc, Mecistops cataphractus; Ts, Tomistoma schlegelii. [file peerj-01-204-s016.png]

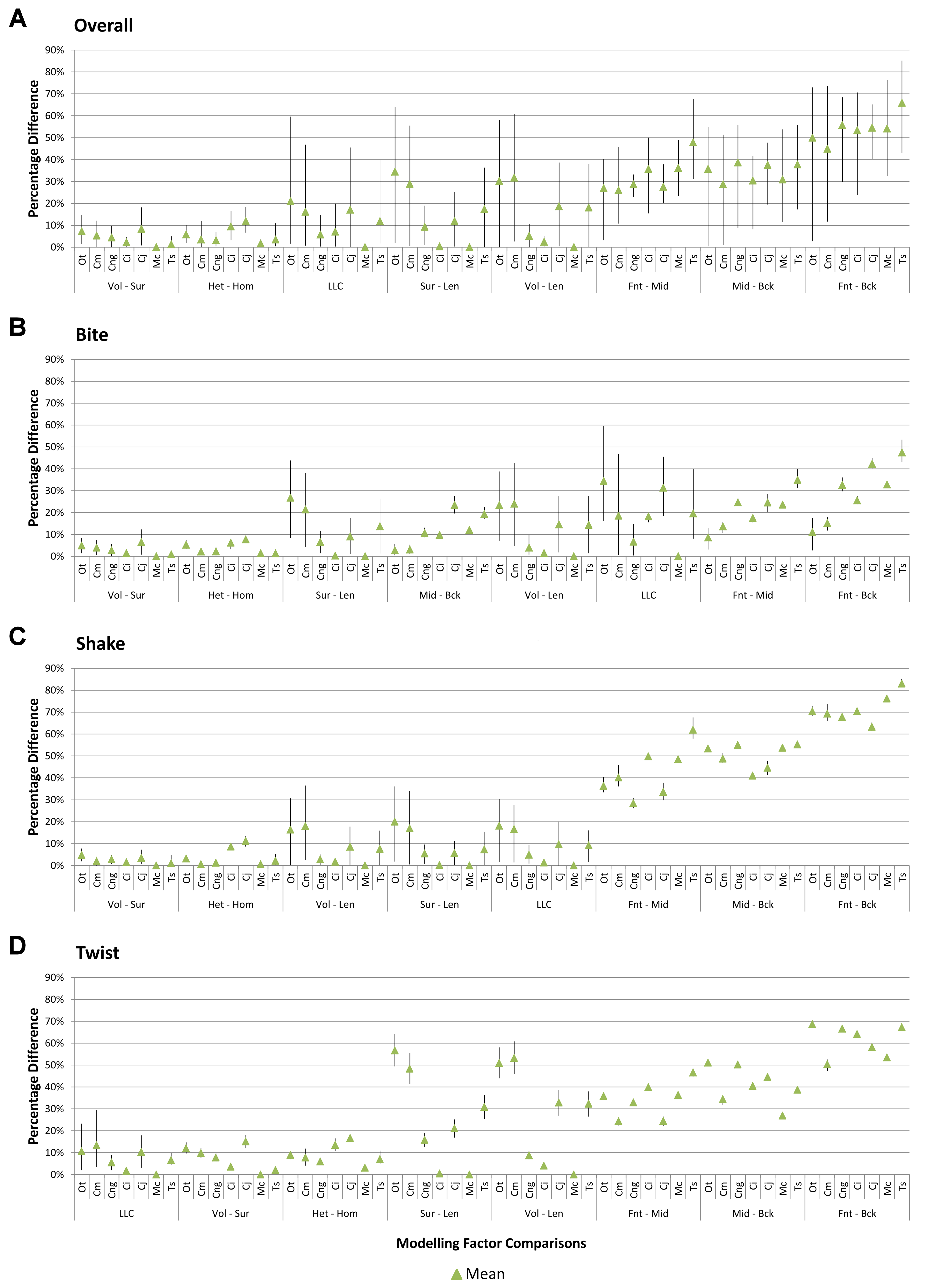

Supplement: Figure S17 — The range of percentage differences for each species is indicated by the upper (maximum % difference) and lower (minimum % difference) extent of vertical bars. These are shown for each of the modelling factor comparisons - which are ordered left to right based on the aggregated average of each comparison. Overall (A) includes differences from all feeding behaviours, while bite (B), shake (C), and twist (D) only include differences from their respective feeding types. Note that the order of modelling factor comparisons changes between biting (B), shaking (C), and twisting (D), suggesting that different feeding types are more (or less) sensitive to different modelling factors. Taxon abbreviations: Ot, Osteolaemus tetraspis; Cm, Crocodylus moreletii; Cng, Crocodylus novaeguineae; Ci, Crocodylus intermedius; Cj, Crocodylus johnstoni; Mc, Mecistops cataphractus; Ts, Tomistoma schlegelii. [file peerj-01-204-s017.png]
