# Supplementary material for: Beware the black box: investigating the sensitivity of FEA simulations to modelling factors in comparative biomechanics
Source: PeerJ. 2013 Nov 5;1:e204. doi: 10.7717/peerj.204 (PMC3828634; doi:10.7717/peerj.204)
Supplement: Table S1 — Reported values for the first two principal components (labelled PC1 and PC2) are from Walmsley et al. (2013). Between them they account for 92% of shape variation within this study group (66% PC1, 26% PC2). ΔPC1 and ΔPC2 are calculated as the difference in shape between each species model (Taxon) to that of Mecistops cataphractus for PC1 and PC2 respectively; these ΔPC scores are effectively a measure of relative difference in the shape of each species to that of Mecistops cataphractus. [file peerj-01-204-s018.docx]

| **Taxon** | **PC1** | **PC2** | **Δ PC1** | **Δ PC2** |
| --- | --- | --- | --- | --- |
| *Osteolaemus tetraspis* | -0.099 | -0.059 | 0.122 | 0.077 |
| *Crocodylus moreletii* | -0.109 | -0.08 | 0.132 | 0.098 |
| *Crocodylus novaeguineae* | -0.04 | 0.048 | 0.063 | 0.03 |
| *Crocodylus intermedius* | -0.009 | 0.049 | 0.032 | 0.031 |
| *Crocodylus johnstoni* | 0.018 | 0.092 | 0.005 | 0.074 |
| *Mecistops cataphractus* | 0.023 | 0.018 | 0 | 0 |
| *Tomistoma schlegelii* | 0.215 | -0.067 | 0.192 | 0.085 |
